# Stabilization of Foxp3 expression by CRISPR-dCas9-based epigenome editing in mouse primary T cells

**DOI:** 10.1186/s13072-017-0129-1

**Published:** 2017-05-08

**Authors:** Masahiro Okada, Mitsuhiro Kanamori, Kazue Someya, Hiroko Nakatsukasa, Akihiko Yoshimura

**Affiliations:** Department of Microbiology and Immunology, Keio University School of Medicine, 35 Shinanomachi, Shinjuku-ku, Tokyo 160-8582 Japan

**Keywords:** Treg, Foxp3, CRISPR, dCas9, TET1, p300, Epigenome editing

## Abstract

**Background:**

Epigenome editing is expected to manipulate transcription and cell fates and to elucidate the gene expression mechanisms in various cell types. For functional epigenome editing, assessing the chromatin context-dependent activity of artificial epigenetic modifier is required.

**Results:**

In this study, we applied clustered regularly interspaced short palindromic repeats (CRISPR)-dCas9-based epigenome editing to mouse primary T cells, focusing on the *Forkhead box P3 (Foxp3)* gene locus, a master transcription factor of regulatory T cells (Tregs). The *Foxp3* gene locus is regulated by combinatorial epigenetic modifications, which determine the Foxp3 expression. Foxp3 expression is unstable in transforming growth factor beta (TGF-β)-induced Tregs (iTregs), while stable in thymus-derived Tregs (tTregs). To stabilize Foxp3 expression in iTregs, we introduced dCas9-TET1CD (dCas9 fused to the catalytic domain (CD) of ten-eleven translocation dioxygenase 1 (TET1), methylcytosine dioxygenase) and dCas9-p300CD (dCas9 fused to the CD of p300, histone acetyltransferase) with guide RNAs (gRNAs) targeted to the *Foxp3* gene locus. Although dCas9-TET1CD induced partial demethylation in enhancer region called conserved non-coding DNA sequences 2 (CNS2), robust Foxp3 stabilization was not observed. In contrast, dCas9-p300CD targeted to the promoter locus partly maintained Foxp3 transcription in cultured and primary T cells even under inflammatory conditions in vitro. Furthermore, dCas9-p300CD promoted expression of Treg signature genes and enhanced suppression activity in vitro.

**Conclusions:**

Our results showed that artificial epigenome editing modified the epigenetic status and gene expression of the targeted loci, and engineered cellular functions in conjunction with endogenous epigenetic modification, suggesting effective usage of these technologies, which help elucidate the relationship between chromatin states and gene expression.

**Electronic supplementary material:**

The online version of this article (doi:10.1186/s13072-017-0129-1) contains supplementary material, which is available to authorized users.

## Background

Epigenetic marks of histone modification and DNA cytosine methylation determine cell identity and function by regulating transcriptional activity at individual loci. Artificial epigenome editing is a novel strategy for manipulating cell fate by altering the specific epigenomic landscape and can help elucidate the mechanisms between chromatin states and gene expression [1]. Epigenome editing tools are fusion proteins consisting of a DNA-binding domain fused with epigenetic-modifying enzymes. Previous reports have shown that DNA-binding proteins, such as zinc finger protein (ZFP) and transcription activator-like effector (TALE) protein, can be used for targeted epigenome editing by fusion with epigenome-modifying enzymes [2, 3]. Although their programmability is verified, there is a disadvantage to designing extensive site-specific constructions. The clustered regularly interspaced short palindromic repeats (CRISPR)-associated protein 9 (Cas9) system (CRISPR-Cas9 system) from *Streptococcus pyogenes* has been used for genome editing by inducing a guide RNA (gRNA) sequence-specific double-strand DNA break. Due to its simple design and high efficiency, the CRISPR-Cas9 system is expected to be utilized extensively in high-throughput and multi-targeted genome editing [4]. Catalytic inactive Cas9 (dCas9) is also recruited to the targeted sequence of the DNA locus, and various fusion proteins with dCas9 can be used for target-specific transcriptional activation and repression [5, 6]. For epigenetic modifications, dCas9 fusion with p300, lysine-specific demethylase 1 (LSD-1), Krüppel-associated box (KRAB), DNA methyltransferase 3a (DNMT3a), and ten-eleven translocation (TET) dioxygenase 1 (TET1) enable gene expression regulation by modifying epigenetic states [7–11]. These biological devices were developed by using cultured cell lines and clearly proposed their versatile performance. However, on the basis that gene transcription is complexly regulated by epigenetic modifications in our body, it is easy to suppose the effectiveness of epigenome editing differs among target loci and cells. Therefore, applying them to primary tissues or cells and evaluation of their activity is expected in the next studies [12]. In primary immune cells, recent research has applied CRISPR-dCas9-based epigenome editing to human primary T lymphocytes, mainly for silencing gene expression [13]. However, only a few studies used epigenome editing mainly for activating gene expression in primary immune cells. Furthermore, little is known about the relationship between artificial epigenome editing and endogenous epigenetic modifications in immune cells.

Regulatory T cells (Tregs) play a pivotal role in regulating immune responses and maintaining immunological tolerance. Treg adoptive transfer therapy is expected to provide a clinical cure for various immunological disorders [14–16]. Tregs are mainly generated via two different routes. The first is through direct development from Treg progenitor cells in the thymus by thymic antigen presentation with high affinity. These Tregs are called naturally occurring Tregs (nTregs) or thymic Tregs (tTregs). The second is through differentiation from naïve CD4 T cells in the periphery by antigen presentation with transforming growth factor (TGF)-β. These Tregs are called induced Tregs in vitro (iTregs) or peripherally induced Tregs (pTregs) [17, 18]. Both Tregs have similar suppression activity and markedly express Forkhead box P3 (Foxp3), a master transcriptional factor for Tregs. Foxp3 expression is required for the differentiation and maintenance of Treg function by expressing Treg signature genes and suppressing effector T cell (Teff) genes [19–23]. The number of available nTregs is limited. It is thought that antigen-specific iTregs could be substituted for nTregs, because iTregs are induced and expanded with antigen specificity in vitro. However, Foxp3 expression is unstable in iTregs owing to the lack of active epigenetic modifications compared with tTregs [24, 25]. Hence, some remaining issues must be resolved prior to the clinical application of ex vivo-expanded iTregs, since iTregs lose Foxp3 expression easily and convert to other pathogenic T cell subsets in vivo [26–28].

The epigenetic modification of the *Foxp3* locus, promoter, and three enhancer regions called conserved non-coding DNA sequences (CNS)1, CNS2, and CNS3, plays pivotal roles in the sustainable expression of Foxp3 [29]. Various transcriptional factors induce active histone modification, such as H3K27 acetylation and H3K4 methylation [30]. Also, the microbial fermentation product butyrate enhances histone acetylation of the *Foxp3* promoter locus and promotes the induction of pTregs in the intestine [31, 32]. In addition to histone modifications, DNA cytosine methylation also effects stable Foxp3 expression. nTregs show a Treg-specific demethylation pattern. Importantly, the *Foxp3* CNS2 locus is also maintained under hypomethylation in nTregs; this hypomethylation contributes to the stable expression of Foxp3 [24, 25, 33]. Recent research has shown that TET family proteins are extensively involved in this demethylation process and maintain Treg stability [34, 35]. In fact, some epigenetic-modifying compounds, such as histone deacetylase (HDAC) inhibitors [36], DNMT inhibitors [37], and TET activators [38], are known for their potential use in effective iTreg induction. However, their target loci are not limited because of low specificity, and there is a risk of undesirable effects like those observed with many of the epigenetic-modifying compounds used to treat cancer [39]. It is essential for functional iTregs to modulate epigenetic modification at necessary locus and not to modulate unnecessary excess locus.

In this study, we established two epigenome-modifying systems based on CRISPR-dCas9 technology and applied them to the *Foxp3* gene locus. We aimed to investigate the cross-talk of epigenome editing and endogenous cellular responses in primary immune cells and to lay a foundation for future clinical development. To stabilize Foxp3 expression in artificially epigenome-edited iTregs: dCas9 fused with TET1CD was targeted to the *Foxp3* CNS2 locus, and dCas9 fused with p300CD to the *Foxp3* promoter locus. We designed 10 gRNA sequences in each locus, screened effective sequences in T cell lines 68-41, and then applied them to mouse primary T cells. We confirmed that both systems with specific gRNAs could induce epigenetic modifications in cultured cell lines. In primary T cells, dCas9-TET1CD partially demethylated the CNS2 locus under iTreg conditions, but Foxp3 expression was not robustly stabilized by inflammatory cytokine stimuli. In contrast, dCas9-p300CD strongly activated and stabilized Foxp3 expression, particularly with TGF-β, even under inflammatory conditions. dCas9-p300CD epigenome-edited iTregs also showed high expression of Treg signature genes and enhanced suppression activity. Through various T cell culture conditions, we concluded that epigenome editing technology can be used in targeted epigenome research, and effectiveness depends on culture conditions. We expect that our study becomes the premise for broad clinical application in the future.

## Methods

### Mice

Foxp3-hCD2-hCD52-KI mice originated from the laboratory of Dr. S Hori (Laboratory of Microbiology, Graduate School of Pharmaceutical Sciences, the University of Tokyo, Tokyo, Japan).

### Antibodies and reagents

For flow cytometry analysis, fluorescein isothiocyanate (FITC), phycoerythrin (PE), peridinin chlorophyll protein-cyanine 5.5 (PerCP-Cy5.5), allophycocyanin (APC), PE-Cy7, and APC-Cy7-conjugated antibodies were purchased from BioLegend (San Diego, CA, USA) or eBioscience (San Diego, CA, USA). The following clones were used: anti-CD4 (RM4-5), Foxp3 (FJK16s), hCD2 (RPA2.10), CD25 (PC61.5), CTLA-4 (UC10-4F10-11), and CD45.1 (A20). Fixable Viability Dye eFluor 780 (FVD780) was used to remove dead cells. Cytokines (mouse interleukin-2 (IL-2), IL-12, IL-4, and IL-6) were purchased from Peprotech (Rocky Hill, NJ, USA), and human TGF-β1 was purchased from BioLegend. LY2157299 was purchased from Shanghai Biochempartner Co., Ltd (Hubei, China).

### Cell culture

Human embryonic kidney cells 293 (HEK293T cells) were obtained from the American Type Culture Collection (ATCC) and maintained in Dulbecco’s modified Eagle medium (DMEM, 4500 mg/l glucose) supplemented with 10% fetal bovine serum (FBS). The 68-41 cells were a gift from Dr. M. Kubo (Division of Molecular Pathology, Research Institute for Biomedical Science, Tokyo University of Science, Tokyo, Japan) [40]. They were maintained in Roswell Park Memorial Institute (RPMI) 1640 medium supplemented with 10% FBS and 55 µM 2-mercaptoethanol (2-ME). Primary T cells were maintained in RPMI 1640 medium supplemented with 10% FBS, 55 µM 2-ME, 1% penicillin/streptomycin, 2 mM l-glutamine, and 100 nM non-essential amino acid solution. All cells were cultured in a humid, 5% CO_2_, 37 °C incubator.

### Plasmid constructions

LentiCRISPR (Addgene, catalog no. 49535) was mutated at amino acid positions D10A and H840A by mutagenesis polymerase chain reaction (PCR) to construct Flag-dCas9-P2A-puro. Mouse TET1 catalytic domain (TET1CD) or p300CD was amplified from complementary DNA (cDNA) and subcloned into a MIGR vector. TET1CD or p300CD and internal ribosome entry site green fluorescent protein (IRES-GFP) sequences were amplified with a Gly–Gly–Gly–Gly–Ser linker and recombined into Flag-dCas9-P2A-puro instead of P2A-puro. Flag-dCas9-TET1CD or p300CD and IRES-GFP were recombined into pMXs-GW vectors. Amino acid sequences of each construct are detailed in the supplementary material (Additional file 1). For gRNA expression, DsRed was recombined into lentiCRISPR instead of Cas9-P2A-puro. Next, U6-gRNA-EFS-DsRed was recombined into CSII vector. Each gRNA expression vector was generated by the annealing of the oligonucleotides, followed by ligation into BsmBI-digested gRNA expression vectors based on CSII vector for lentiviral infection. In some cases, U6-gRNA-EFS-DsRed was recombined into pMXs-GW vectors for retroviral infection. gRNA off-target predictions were performed by CCTop—CRISPR/Cas9 target online predictor [41]. Max. total mismatches, core length, and max. core mismatches were set to 4, 12, and 2, respectively. Predicted off-target loci were listed in Additional file 2: Table S1.

### Retroviral or lentiviral production

pMXs and pCL-Eco were co-transfected for retroviral production, or CSII, pMDLg/pRRE, and VSV-G/Rsv-Rev were co-transfected for lentiviral production into HEK293T cells using polyethylenimine MAX (PEI-MAX), followed by a medium change to remove the transfection reagents. Virus-containing medium was harvested, filtered (0.45 µm), and then concentrated by centrifugation overnight.

### Establishment of dCas9-TET1CD or p300CD stable 68-41 cells and gRNA transduction

dCas9-TET1CD- or p300CD-expressing retrovirus was transduced into 68-41 cells with 5 µg/ml polybrene with centrifugation at 2500 rpm for 2 h at 35 °C. GFP-positive cells were sorted using a SH800 (SONY, Tokyo, Japan) or ARIA (BD Biosciences, San Jose, CA, USA) cell sorter. GFP-positive cells were further cultured and maintained GFP-positive >85% by sorting again. These stable cell lines were roughly isolated and not from a single clone. Next, gRNA-expressing lentivirus was transduced as above, and further analysis was performed. Transduction efficiency was almost >80%, but in the case of low transduction efficiency, GFP and DsRed double-positive cells were sorted. For intracellular Foxp3 staining, fixation buffer (eBiosciences) and 0.2% Triton-X were used for fixation and permeabilization to retain GFP and DsRed fluorescence.

### Primary T cell culture

Naïve CD4+ T cells (CD4+CD25-hCD2-CD62L+) were isolated using magnetic-activated cell sorting (MACS) from the spleen and lymph nodes of 6- to 8-week-old-male Foxp3-hCD2-hCD52-KI mice. Splenocytes and lymphocytes were red blood lysed and depleted using AutoMACS (Miltenyi Biotec, Tokyo, Japan) with biotin-conjugated anti-B220 (RA3-6B2), CD8a (53-67), CD49b (DX-5), CD11b (M1/70), CD11c (N418), CD25 (PC61), TER119 (TER-119), Ly6G (RB6-8C5), T cell receptor (TCR)-γδ (GL3), and sometimes hCD2 (RPA2.10) (BioLegend or eBioscience), or CD4 isolation kit (Miltenyi Biotec) with anti-CD25 and sometimes hCD2, and then with anti-Biotin microbeads or streptavidin microbeads (Miltenyi Biotec). CD4+ T cells were further incubated with CD62L microbeads (Miltenyi Biotec), and CD4+CD62L+ T cells were positively selected by using AutoMACS. The purity was almost >95%. Isolated naïve CD4+ T cells (3–4 × 10^5^ cells) were cultured on 24-well plates under iTreg conditions using anti-CD3e (plate-coated 2C11, 4 µg/ml), anti-CD28 (PV1.17.10, 1.2 µg/ml), anti-IFN-γ (R4-6A2, 5 µg/ml), anti-IL-4 (11B11, 5 µg/ml), recombinant human TGF-β (2 ng/ml), and IL-2 (20 ng/ml). On day 2, dCas9-TETCD or p300CD and gRNA were co-transduced with 6 µg/ml polybrene (Merck Millipore, Billerica, MA, USA) or 10 µg/ml Protransduzin A (Immundiagnostik AG, Bensheim, Germany) (mainly for sorting experiment), with centrifugation at 2500 rpm for 2 h at 35 °C. The next day, co-transduced iTregs were harvested and further cultured for 2 days under Th1, Th2, and Th0 + IL-6 conditions using anti-CD3e (plate-coated, 4 µg/ml), anti-CD28 (1.2 µg/ml), and IL-2 (20 ng/ml). Conditions were as follows: Th1, anti-IL-4 (5 µg/ml), IL-12 (20 ng/ml); Th2, anti-IFN-γ (5 µg/ml), IL-4 (20 ng/ml); Th0 + IL-6, anti-IFN-γ (5 µg/ml), anti-IL-4 (5 µg/ml), IL-6 (20 ng/ml); Th0, anti-IFN-γ (5 µg/ml), anti-IL-4 (5 µg/ml).

For helper T cell subsets skewing, naive CD4+ T cells (3–4 × 10^5^ cells) were cultured on 24-well plates under Th1, Th2, and Th17 conditions. Th1 and Th2 were the same as above: Th17, anti-IFN-γ (5 µg/ml), anti-IL-4 (5 µg/ml), human TGF-β (0.5 ng/ml), IL-6 (20 ng/ml).

For TGF-β signal inhibition experiment, indicated concentration of TGF-β was used for iTreg condition in the presence of LY2157299 or anti-TGF-β (1D11).

For intracellular cytokine staining, cells were stimulated with 50 ng/ml phorbol myristate acetate (PMA), 1 µg/ml ionomycin, and Brefeldin A solution for 4 h. Cells were harvested and stained with FVD780, anti-CD4, hCD2, and CD25. For intracellular staining, anti-IFN-γ, anti-IL-2, and anti-CTLA-4 were used after fixation buffer and permeabilization buffer (eBioscience).

### Western blot analysis

The cells were lysed using an immunoprecipitation lysis buffer (50 mM Tris–HCl (pH 7.5), 150 mM NaCl, 10 mM ethylenediaminetetraacetic acid (EDTA, pH 8.0), 1% sodium deoxycholate, 1% Triton X-100, 5 μg/ml leupeptin, and 1 mM PMSF). The cell lysates were centrifuged, and the supernatants were mixed with 5*sodium dodecyl sulfate (SDS) sample buffer (10% SDS, 40% glycerol, 0.2 M Tris–HCl (pH 6.8), 0.025% bromophenol blue, and 50 mM dithiothreitol [DTT]). After boiling, the samples were separated through electrophoresis and transferred to polyvinylidene difluoride (PVDF) membranes. The membranes were probed with anti-Flag (M2; Sigma-Aldrich, St. Louis, MO, USA) and anti-α-tubulin (DM1A; Sigma-Aldrich) and detected using the Chemi-Lumi One system (Nacalai Tesque, Kyoto, Japan).

### Bisulfite sequencing

Cells were lysed using Wizard SV Lysis Buffer (Promega Corporation, Madison, WI, USA). Genomic DNAs were isolated by phenol–chloroform extraction, isopropanol precipitation, and 70% ethanol purification. Genomic DNAs were digested with BamHI, and the same amount of digested DNA (<1.5 µg) was aligned to 19 µl by adding H_2_O. Next, 1.2 µl of 5 M NaOH was added and incubated at 37 °C for 15 min. Then, 121.2 µl of bisulfite mixture was added and incubated for 1 h at 80 °C. The bisulfite mixture (121.2 µl) consisted of 3.6 M sodium bisulfite (1.92 g in 4.4 ml H_2_O; 107 µl), 0.57 mM hydroquinone (11 mg in 10 ml H_2_O; 7 µl), and 0.3 M NaOH (5 M NaOH; 7.2 µl). After purification and extraction using a 50-µl GP3 solution from a column using a FastGene Gel/PCR Extraction Kit (Nippon Genetics, Tokyo, Japan), 3 µl of 5 M NaOH was added and incubated for 5 min at 37 °C to complete the bisulfite reaction. Bisulfite products were precipitated using isopropanol with 10 µg glycogen and rinsed using 70% ethanol. The Foxp3 CNS2 locus was amplified by Quick Taq HS DyeMix (Toyobo Life Science, Tokyo, Japan) polymerase with a bisulfite sequence primer (forward primer: TTTTGGGTTTTTTTGGTATTTAAGA and reverse primer: AACTAACCAACCAACTTCCTACACTAT designed by MethPrimer [online]) and then subcloned into pGEM-T EASY Vector (Promega Corporation). Plasmids were purified and sequenced using SP6 primer. Methylation analysis was performed using the quantification tool for methylation analysis (online).

### Real-time PCR analysis

Cells were lysed using RNAiso Plus (Takara Bio Inc., Shiga, Japan). Total RNA was isolated by chloroform extraction, isopropanol precipitation, and 70% ethanol purification. Total RNA from primary T cells was isolated by using ReliaPrep RNA Miniprep Systems (Promega Corporation) after treatment with RNase inhibitor from human placenta (Nacalai Tesque). cDNA was synthesized by reverse transcription by a High-Capacity cDNA Synthesis Kit (Applied Biosystems, Thermo Fisher Scientific K.K., Kanagawa, Japan) from RNA. Real-time PCR (RT-PCR) analysis was performed using an iCycler iQ multicolor RT-PCR detection system with SsoFast EvaGreen Supermix (Bio-Rad Laboratories, Hercules, CA, USA). Amplification primers (mFoxp3, forward primer: CCCAGGAAAGACAGCAACCTT, reverse primer: TTCTCACAACCAGGCCACTTG, mHPRT1, forward primer: TGAAGAGCTACTGTAATGATCAGTC, reverse primer: AGCAAGCTTGCAACCTTAACCA) were used for quantification.

### Chromatin immunoprecipitation assay

Cells were fixed with 1 ml of 1% formaldehyde for 10 min at room temperature and washed two times using phosphate-buffered saline (PBS). Then, cells were lysed using lysis buffer (1% SDS, 10 mM EDTA (pH 8.0), 50 mM Tris–HCl (pH 8.0), and protease inhibitor cocktail (Nacalai Tesque)). Genomes were sonicated to a mean size of 300 bp using an Acoustic Solubilizer (Covaris, Woburn, MA, USA) with the “300 bp shearing pro” program. Genomic fragment solutions were diluted tenfold with chromatin immunoprecipitation (ChIP) dilution buffer (0.01% SDS, 1.1% Triton-X, 1.2 mM EDTA (pH 8.0), 16.7 mM Tris–HCl (pH 8.0), 167 mM NaCl, and protease inhibitor cocktail). The input samples were collected, and the remainders were immunoprecipitated with 1 µg of Anti-acetyl-Histone H3 Antibody (Merck Millipore) or control rabbit immunoglobulin (Ig) G (Santa Cruz Biotechnology, Inc, Dallas, TX, USA), followed by incubation with dynabeads protein G (Invitrogen, Thermo Fisher Scientific K.K.). Precipitants were washed in order, using low salt wash buffer (0.1% SDS, 1% Triton-X, 2 mM EDTA (pH 8.0), 20 mM Tris–HCl (pH 8.0), and 150 mM NaCl), high salt wash buffer (0.1% SDS, 1% Triton-X, 2 mM EDTA (pH 8.0), 20 mM Tris–HCl (pH 8.0), and 500 mM NaCl), LiCl buffer [0.25 M LiCl, 1% NP-40, 1% sodium deoxycholate, 1 mM EDTA (pH 8.0), and 10 mM Tris–HCl (pH 8.0)], and TE buffer (twice). Chromatin samples were eluted twice by incubation with 100 µl of elution buffer (1% SDS, 0.1 M NaHCO_3_, and 10 mM DTT) for 15 min at room temperature. Followed by de-cross-linking using incubation at 65 °C overnight, 8 µl of 5 M NaCl was added to elution products. Next, 20.36 µl proteinase K mixture was added and incubated at 45 °C for 6 h. The proteinase K mixture (20.36 µl) consisted of 0.5 M EDTA (pH 8.0): 4 µl, 0.5 M Tris–HCl (pH 6.8): 16 µl, 20 mg/ml proteinase K: 0.36 µl. Purification and extraction were performed using a 50-µl GP3 solution from a column using a FastGene Gel/PCR Extraction Kit (Nippon Genetics). ChIP products were analyzed by quantitative PCR. Amplification primers (forward primer: CCCTGCAATTATCAGCACACAC, reverse primer: ATCAGCCTGGCTTGTGGGAAAC) were used for quantification.

### In vitro Treg suppression assay

Responder effector cells (CD4+CD25-) isolated by MACS as described above from Ly5.1 cognate mice were labeled with 2 µM carboxyfluorescein diacetate succinimidyl diester (CFSE) in 37 °C PBS for 10 min and then washed with sufficient RPMI medium, termed as Teff. Splenic CD11c-positive cells isolated by CD11c beads (Miltenyi Biotec) were used as antigen-presenting cells. iTregs co-transduced with dCas9-p300CD and gRNAs, gated on CD4+GFP+DsRed+hCD2+ cells, were sorted using a cell sorter SH800. Next, 4 × 10^4^ Teff and 2 × 10^4^ splenic dendritic cells (DCs) with 1 µg/ml soluble anti-CD3e, with or without 1 × 10^4^ iTreg, were cultured in a 96-well U-bottomed dish for 4 days. CFSE dilution and Foxp3 (hCD2) expression were analyzed.

### Statistical analysis

All values are presented as the means ± standard deviations (SDs). Unpaired Student’s *t* tests were used, and *p* < 0.05 was defined as statistically significant.

## Results

### Constructions and expression

**Fig. 1:**
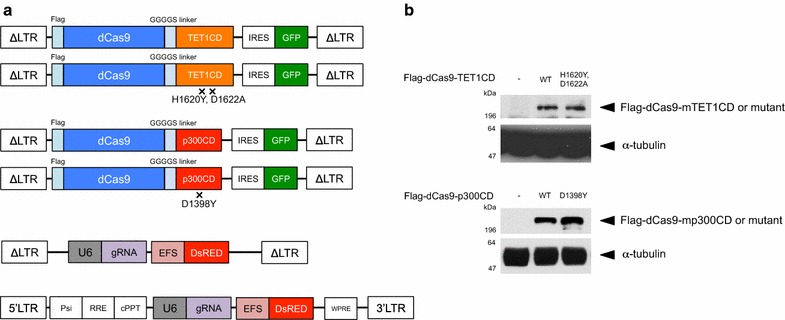
CRISPR-dCas9-based epigenome editing for primary T cells. **a** A retroviral vector for the expression of dCas9-epigenome regulator fusion proteins from Moloney murine leukemia virus promoter long terminal repeats (ΔLTRs) and green fluorescent protein (GFP) from an internal ribosomal entry site (IRES). Retroviral and lentiviral vector for bicistronic expression of the gRNA from a U6 promoter (U6) and DsRed from a short EF1a promoter (EFS). **b** Protein expression of dCas9-epigenome regulator fusion proteins in transfected HEK293T cells was detected by western blot against anti-Flag antibody. Anti α-tubulin antibody was used for loading control

For targeted epigenome editing, we constructed a retroviral expression system for dCas9-TET1CD and dCas9-p300CD fusion proteins. Mouse TET1CD H1620Y, D1622A, and mouse p300CD D1398Y were mutated for catalytic inactive mutants. As these retroviral vectors contain the IRES-GFP sequence, the expression of fusion proteins can be monitored by GFP expression. For gRNA expression, we constructed retroviral or lentiviral expression systems that contain DsRed as the fluorescence marker (Fig. 1a). We confirmed the expression of fusion proteins in HEK293T cells using western blotting methods (Fig. 1b).

### dCas9-TET1CD induced demethylation of the Foxp3 CNS2 locus, but weakly sustained Foxp3 expression

**Fig. 2:**
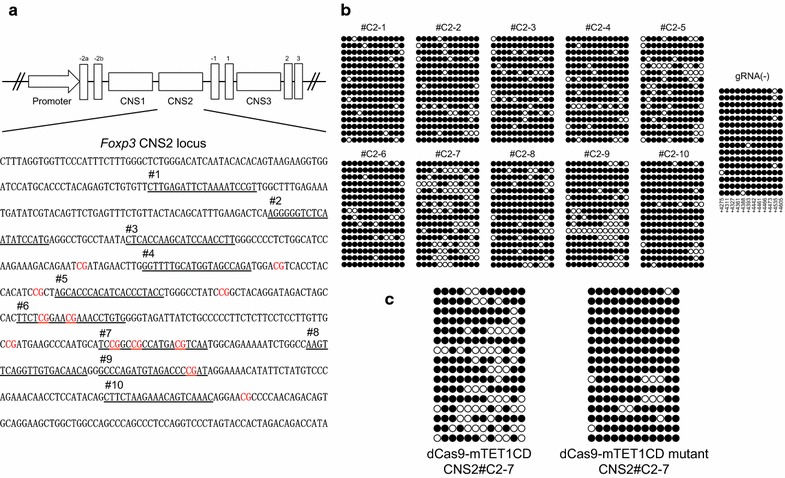
dCas9-TET1CD-mediated demethylation of the *Foxp3* CNS2 locus. **a** Sequence at the *Foxp3* CNS2 locus is shown. Each gRNA sequence is *underlined* and *numbered* #C2-1 to #C2-10. Specific CpG sites are *lettered red*. **b** and **c** The methylation status of CpG sites at the *Foxp3* CNS2 locus in dCas9-TET1CD and each gRNA-expressing 68-41 cells (**b**) and dCas9-TET1CD or the TET1CD catalytic mutant and gRNA #C2-7 expressing 68-41 cells (**c**) was determined by bisulfite sequence analysis. The 68-41 cells stably expressing dCas9-TET1CD were transduced with each gRNA expression lentivirus and sorted (**b**). The 68-41 cells were co-transduced with dCas9-TET1CD or TET1CD mutant and gRNA CNS2#C2-7 and sorted (**c**). A *horizontal row* depicts one sequenced clone in which CpGs was methylated (*black*) or demethylated (*white*). Data are pooled from two independent experiments

The *Foxp3* CNS2 locus contains 12 CpG sites, and its methylation or demethylation status is extensively involved in the unstable or stable Foxp3 expression phenotype, respectively. To edit the methylation status, we designed 10 gRNA sequences at the *Foxp3* CNS2 locus (Fig. 2a) and transduced them into a 68-41 T cell line that stably expressed dCas9-TET1CD. The *Foxp3* CNS2 locus was heavily methylated in the 68-41 T cell line. After purifying gRNA-positive cells, the methylation status of the *Foxp3* CNS2 locus was analyzed by bisulfite sequencing. The results revealed that several gRNA sequences, such as #C2-7 and #C2-5, could induce demethylation to some extent (approximately 30% by #C2-7), whereas #C2-1 and #C2-10 had little effect (Fig. 2b). The catalytic inactive mutant of TET1CD induced less with #C2-7, indicating that demethylation by dCas9-TET1CD was TET enzyme activity dependent (Fig. 2c). We selected #C2-1 as a negative control gRNA and #C2-7 as a positive control gRNA. Unlike the reported demethylation pattern by TALE-TET1 fusion proteins [42], dCas9-TET1CD fusion proteins could demethylate some CpG sites distant from the designed gRNA sequences. These findings coincide with previous reports [11, 43].

**Fig. 3:**
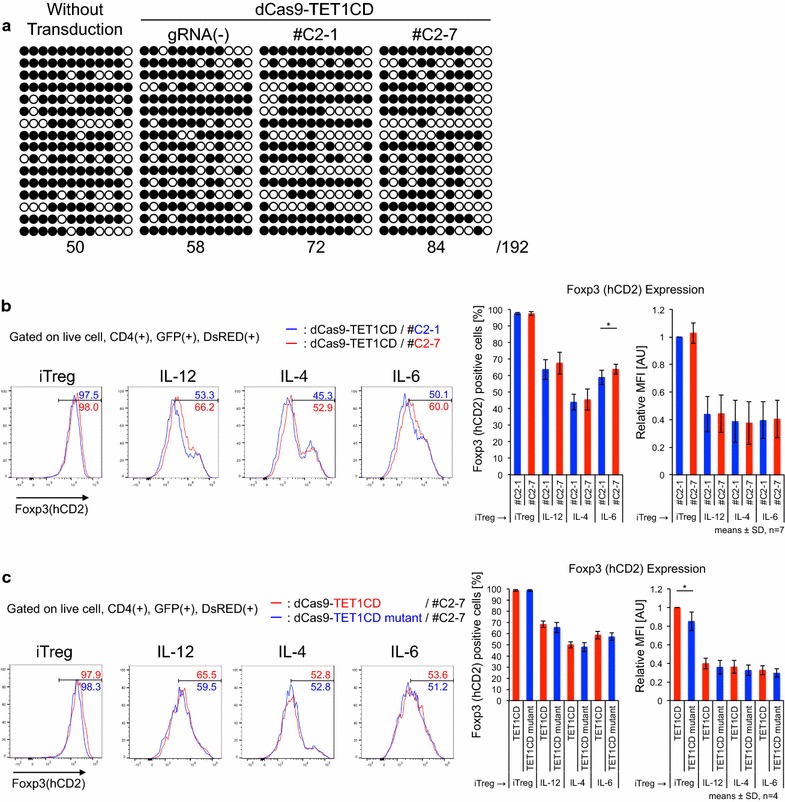
Maintenance of Foxp3 expression induced by dCas9-TET1CD-mediated demethylation of the *Foxp3* CNS2 locus. **a** The methylation status of CpG sites at the *Foxp3* CNS2 locus in untransduced, dCas9-TET1CD single, with #C2-1, and with #C2-7 transduced iTregs was determined by bisulfite sequence analysis. GFP/DsRed(−/−), (+/−), and (+/+) cells are sorted, respectively. A *horizontal row* depicts one sequence clone in which CpGs was methylated (*black*) or demethylated (*white*). The *number* below bisulfite sequences indicates demethylated CpG sites. Data are pooled from two independent experiments. (**b** and **c**) Flow cytometry analysis of Foxp3(hCD2) expression in iTregs co-transduced with dCas9-TET1CD and gRNA CNS2 #C2-1 (*blue*) or #C2-7 (**red**) (**b**), dCas9-TET1CD (***red***), or TET1CD mutant (*blue*) and gRNA #C2-7 (**c**) under inflammatory cytokine conditions. Percentages of Foxp3(+) and mean fluorescence intensity (MFI) relative value to iTregs co-transduced with dCas9-TET1CD and #C2-1 (**b**), dCas9-TET1CD and #C2-7 (**c**) were plotted. Data are pooled from seven (**b**) or four (**c**) independent experiments and represent the means ± SDs. *p < 0.05

Next, we applied dCas9-TET1CD to primary T cells from male Foxp3-hCD2-hCD52-KI mice under iTreg skewing conditions and confirmed its demethylation activity in iTregs. Unlike 68-41 T cell lines, iTregs showed slight demethylation at the CNS2 locus at the basal level in the absence of gRNAs, and this demethylation was enhanced by co-transduction of dCas9-TET1CD with gRNAs (Fig. 3a). We then examined the promotive effect of dCas9-TET1CD on Foxp3 stability in iTregs. Foxp3 stability under inflammatory conditions was investigated using the following method. Naive CD4+ T cells were cultured under iTreg skewing conditions for 3 days, resulting in >90% Foxp3(+) cells. iTregs were harvested and further cultured under the same iTreg conditions (for positive control) or under inflammatory cytokine (in the presence of IL-12, IL-4, or IL-6) conditions for 2 days. This re-stimulation destabilized Foxp3 expression, which was monitored by surface hCD2 staining correlated with intracellular Foxp3 staining as shown in Additional file 3: Figure S1. The results coincide with previous reports [44–47]. To retain GFP and DsRed fluorescence, we monitored Foxp3 expression by hCD2 without intracellular staining. Using this method, compared with no gRNA-transduced cells (GFP(+)DsRed(−) cells), dCas9-TET1CD and gRNA co-transduction yielded stabilized Foxp3 expression (Additional file 3: Figure S2a). Since demethylation occurred in #C2-1 co-transduced iTregs to some extent, a partial stabilization effect was observed in #C2-7 co-transduced iTregs in comparison with #C2-1 co-transduced iTregs (Fig. 3b), which were confirmed by *Foxp3* mRNA expression (Additional file 3: Figure S2b). Comparable to the dCas9-TET1CD mutant, similar stabilization effects were detected to some extent (Fig. 3c). These data indicated that dCas9-TET1CD for the *Foxp3* CNS2 locus had a certain stabilizing effect for Foxp3 expression, but its effect was weak especially when exposed to inflammatory cytokines (Fig. 7a). A previous report suggested that inflammatory cytokine signals (IL-4/STAT6, IL-6/STAT3) recruit DNMT1 and DNMT3a to the CNS2 locus after stimulation, leading to Foxp3 loss even in nTregs [46]. We speculated that dCas9-TET1CD targeted to the CNS2 locus competes with methyltransferases under inflammatory conditions, resulting in earlier loss of demethylation function than under iTreg conditions. In fact, Foxp3 mean fluorescence intensity (MFI) was greater in dCas9-TET1CD than in TET1CD catalytic inactive mutant under iTreg conditions (Fig. 3c), but was weakened by inflammatory stimuli.

### dCas9-p300CD induced acetylation of the Foxp3 promoter locus and induced stable expression of Foxp3 in a cultured T cell line

**Fig. 4:**
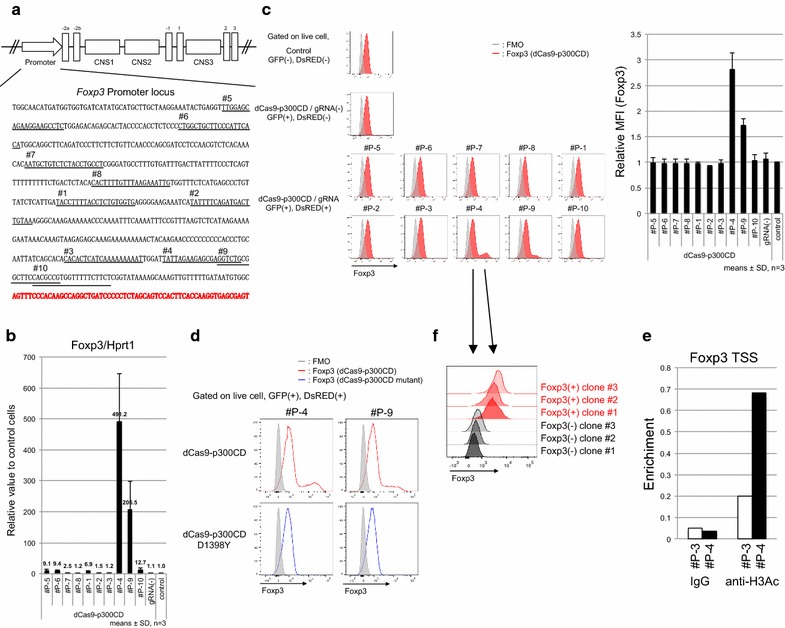
dCas9-p300CD-mediated *Foxp3* promoter acetylation and transcriptional activation. **a** Sequence at the *Foxp3* promoter locus is shown. Each gRNA sequence is *underlined* and *numbered* #P-1 to #P-10. Transcription start sites are *lettered bold red*. **b** Foxp3 mRNA expression in each gRNA-transduced 68-41 cell stably expresses dCas9-p300CD relative to control 68-41 cells (control). Data are pooled from three independent experiments and represent the means ± SDs. **c** Flow cytometry analysis of Foxp3 expression in each gRNA-transduced 68-41 cell stably expresses dCas9-p300CD. Foxp3 MFI relative value to control 68-41 cells was plotted. Data are pooled from three independent experiments and represent the means ± SDs. **d** Flow cytometry analysis of Foxp3 expression in each gRNA-transduced 68-41 cell stably expresses dCas9-p300CD or p300 mutant. **e** Enrichment of acetyl histone H3 at *Foxp3* TSS locus in #P-3 or #P-4 transduced 68-41 cell stably expresses dCas9-p300CD. **f** Flow cytometry analysis of Foxp3 expression in each clone isolated by limiting the dilution from dCas9-p300CD and #P-4 co-transduced 68-41 cells

Foxp3 expression is induced by histone acetylation of the promoter locus. We designed 10 gRNA sequences at the *Foxp3* promoter locus (Fig. 4a) and transduced them into the 68-41 T cell line that stably expressed dCas9-p300CD. The 68-41 cell line showed little Foxp3 expression. We measured the amount of mRNA expression induced by dCas9-p300CD. We observed that #P-4 and #P-9 strongly activated Foxp3 transcription, and that #P-5, #P-6, #P-1, and #P-10 induced moderately activated Foxp3 transcription (Fig. 4b). Next, we assessed protein expression. A small but significant fraction of endogenous Foxp3 expression could be detected in #P-4 and #P-9 transduced cells (Fig. 4c). This induction was dependent on p300CD autoacetylation activity, since the catalytic inactive mutant could not induce it (Fig. 4d). We selected #P-3 as a negative control gRNA and #P-4 as a positive control gRNA. In #P-4 transduced cells, the histone acetylation of the Foxp3 promoter locus was promoted compared with #P-3, correlating with transcriptional activation (Fig. 4e).

Although similar expression levels of dCas9-p300CD and #P-4 are gated, approximately 10% of the population significantly expressed Foxp3. By limiting dilution, we isolated several clones that stably expressed a high amount of Foxp3, and others that never expressed it (Fig. 4f). Although we could not explain the mechanism of this bipolarization phenomenon, the data suggested that it was not due to the oscillation of the cell population. The data indicated that targeted histone acetylation could strongly maintain epigenetic modification and transcriptional activation in certain specific cells.

### dCas9-p300CD induces stable Foxp3 expression in primary T cells

**Fig. 5:**
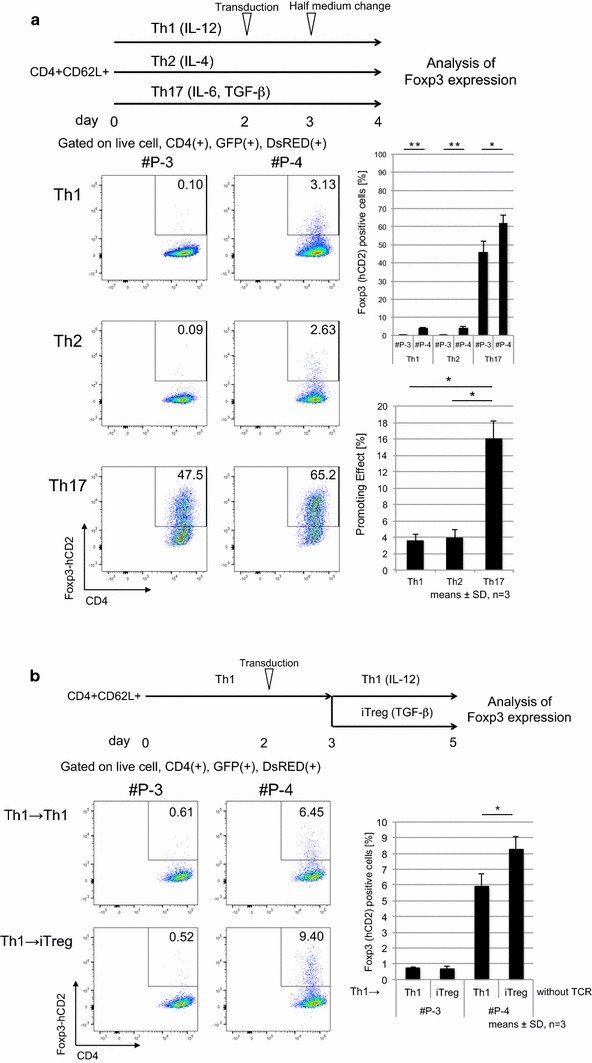
dCas9-p300CD-mediated Foxp3 transcriptional activation is strengthened by TGF-β signal. **a** Upper, experimental scheme. Magnetic-activated cell sorting (MACS)-sorted naïve CD4+ T cells were cultured under Th1, Th2, and Th17 skewing conditions. On day 2, dCas9-p300CD and gRNA #P-3 or #P-4 were transduced with polybrene and further cultured for 2 days. Lower, flow cytometry plots of Th subsets co-transduced with dCas9-p300 and gRNAs show expression of CD4 and Foxp3 (hCD2). Percentages of Foxp3(+) cells and subtraction of #P-3 from #P-4 were plotted. Data are pooled from three independent experiments and represent the means ± SDs. *p < 0.05; **p < 0.01. **b** Upper, experimental scheme. MACS-sorted naïve CD4+ T cells were cultured under Th1 skewing conditions, and on day 2, dCas9-p300CD and gRNAs were transduced with polybrene. The next day, Th1 was harvested and further cultured under Th1 or iTreg skewing conditions without T cell receptor (TCR) stimulation. Lower, flow cytometry plots of Th1 co-transduced with dCas9-p300CD and gRNAs show expression of CD4 and Foxp3 (hCD2). Percentages of Foxp3(+) cells were plotted. Data are pooled from three independent experiments and represent the means ± SDs. *p < 0.05; **p < 0.01

We applied this system to primary T cells. Under helper T cell culture skewing conditions, we co-transduced dCas9-p300CD and #P-3 and #P-4 into isolated naïve CD4+ T cells and investigated Foxp3 expression. Foxp3 expression was induced under all skewing conditions, and a notably superior enhancing effect was observed under the Th17 condition (IL-6 and TGF-β), as shown in Fig. 5a. IL-2 was added to all skewing conditions to improve T cell proliferation and transduction efficiency. Thus, the majority of the population expressed Foxp3, even under Th17 conditions. TGF-β induces Foxp3 mainly through the *Foxp3* CNS1 enhancer locus by Smad2 and Smad3 signals [29, 48, 49]. This indicates that the artificial histone acetylation of the promoter locus activated transcription from the promoter locus, and it was augmented by the TGF-β signal, which principally activates the enhancer locus. In other words, the TGF-β signal additionally activated Foxp3 transcription, even when the promoter locus was artificially opened. This possible enhanced activity of dCas9-p300CD under TGF-β signal condition was confirmed by blocking its signals. Low-dose TGF-β enhanced Foxp3 expression within #P-4 co-transduced cells than #P-3, and this effect was cancelled by treating LY2157299 (TGF-β receptor kinase inhibitor) or anti-TGF-β antibody, indicating the involvement of TGF-β signal in enhancing dCas9-p300CD activity (Additional file 3: Figure S3). Then, we speculated whether co-transduction of dCas9-p300CD and #P-4 facilitated TGF-β signal to engage in transactivation by investigating Foxp3 expression in T cell plasticity culture (Th1 to iTregs). Since helper T cell subset plasticity is strictly regulated, Foxp3 cannot be induced by TGF-β in already differentiated Th1 cells [50]. As we expected, Foxp3 expression was promoted in dCas9-p300CD and #P-4 co-transduced cells, even when differentiated Th1 cells were further treated with TGF-β. This promotion effect never occurred in #P-3 cells (Fig. 5b). Epigenome editing could thus be a novel method for converting the T cell subset.

**Fig. 6:**
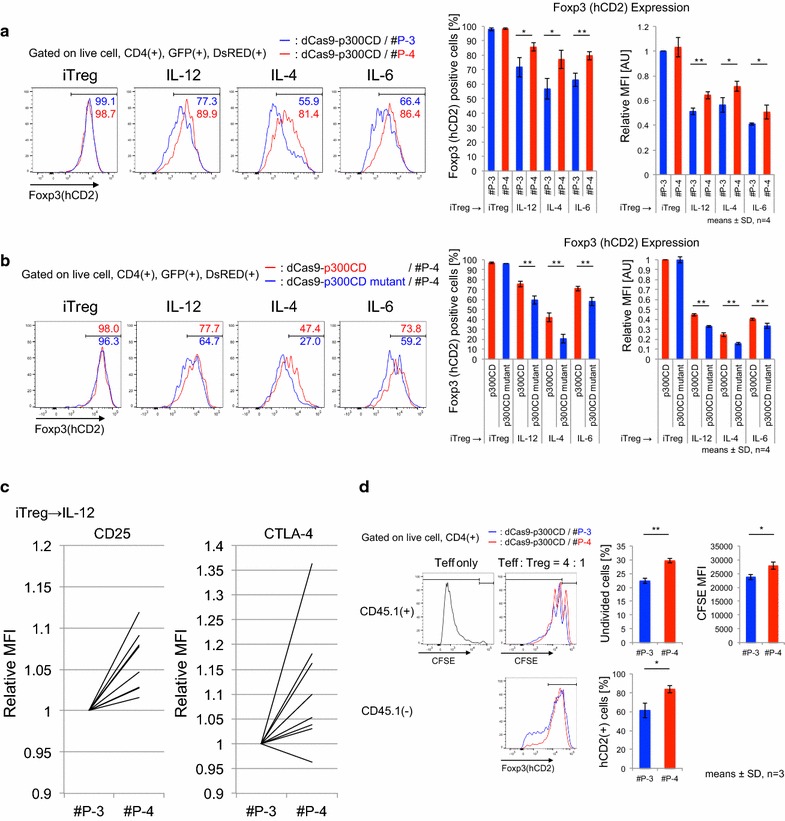
dCas9-p300CD-applied iTregs showed higher suppressive activity. **a** and **b** Flow cytometry analysis of Foxp3 (hCD2) expression in iTregs co-transduced with dCas9-p300CD and gRNA #P-3 (*blue*) or #P-4 (*red*) (**a**), dCas9-p300CD (*red*) or p300CD mutant (*blue*) and #P-4 (B). Percentages of Foxp3(+) cells and MFI relative value to iTregs co-transduced with dCas9-p300CD and #P-3 (**a**) or dCas9-p300CD and #P-4 (**b**) were plotted. Data are pooled from four independent experiments and represent the means ± SDs. *p < 0.05; **p < 0.01. **b** Relative expression of CD25 or CTLA-4 of IL-12-treated iTregs co-transduced with dCas9-p300CD and gRNAs. Relative MFI values to #P-3 were plotted. Data represent each experimental value of eight independent experiments. **c** Treg suppression activity was measured by CFSE dilution in labeled Teff. Flow cytometry analysis of CFSE dilution in labeled Teff, co-cultured with or without iTregs co-transduced with dCas9-p300CD and gRNA #P-3 (*blue*) or #P-4 (*red*). Foxp3 (hCD2) expression in iTregs co-transduced with dCas9-p300CD and gRNA #P-3 (*blue*) or #P-4 (*red*) was also analyzed. Data are pooled from three independent experiments and represent the means ± SDs. *p < 0.05; **p < 0.01

**Fig. 7:**
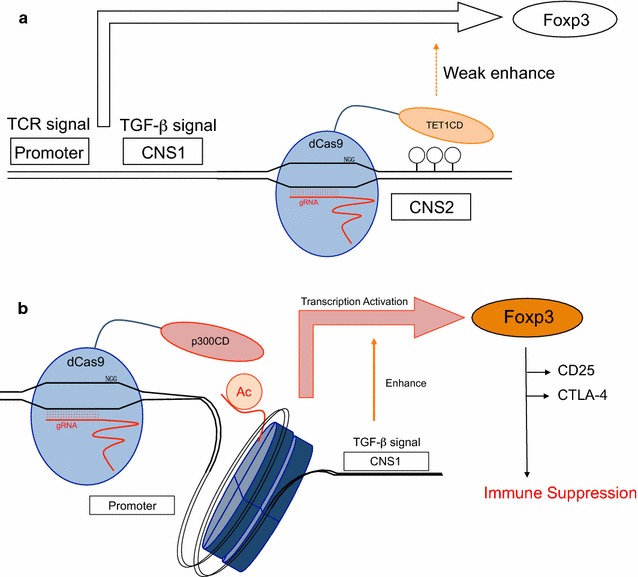
Model of epigenome editing in primary T cells. **a** dCas9-TET1CD demethylates the *Foxp3* CNS2 locus and enhances Foxp3 expression weakly. **b** dCas9-p300CD acetylates the *Foxp3* promoter locus, activates transcription in coordination with the TGF-β signal, and promotes immunosuppressive function

Then, we examined the maintenance of Foxp3 by dCas9-p300CD in iTregs. The stability of Foxp3 under inflammatory cytokines was investigated (same as Additional file 3: Figure S1). We observed that iTregs co-transduced with dCas9-p300CD and #P-4 retained a high amount of Foxp3 compared with #P-3 under inflammatory conditions (Fig. 6a). We confirmed this maintenance is actually dependent on p300CD autoacetylation activity by co-transduction with p300 catalytic inactive mutant (Fig. 6b). Moreover, the Treg signature genes CD25 and CTLA-4 were slightly but significantly upregulated under IL-12 conditions in #P-4 transduced iTregs (Fig. 6c). Finally, we examined the iTreg suppression activity in vitro. iTregs co-transduced with dCas9-p300CD and gRNA were sorted (Additional file 3: Figure S4a). Splenic DCs were used as antigen-presenting cells, and the proliferation of effector T cells was further suppressed by #P-4 transduced iTregs, which correlated with Foxp3 stabilization (Fig. 6d). Similar tendency was observed in comparison with catalytic activity (Additional file 3: Figure S4b). These data showed that applying dCas9-p300CD to primary T cells, especially iTregs, could modify both transcription and cell function. These data also clarified one aspect of the Foxp3 transcriptional activation mechanisms (Fig. 7b).

### Off-target analysis of selected gRNAs

CRISPR-Cas9 or CRISPR-dCas9-based technologies are constantly at risk of off-target activity [51, 52]. For clinical usage or to validate results, we have to consider off-target effects. We used the CCTop online tool [41], and selected gRNA sequences (#C2-1, #C2-7, #P-3, and #P-4) were investigated for potential off-target sites. We observed that the selected gRNA sequences had at least three mismatches on similar sequences, and most off-target candidate sequences were localized in intergenic regions (Additional file 2: Table S1). In our study, which mainly focused on mice experiments and revealing the relationships between epigenetics and gene expression, all candidate genes were not strongly involved in direct Foxp3 induction or Treg functions to the best of our knowledge. For future clinical usage, we have to re-select gRNA sequences in the human genome and investigate off-target activity in our next study.

## Discussion

Artificial targeted epigenome editing mediated by CRISPR-dCas9 can be utilized to clarify the relationship between chromatin states and gene expression and to develop novel clinical strategies. Previous research proposed this biological device and demonstrated its universal performance and efficiency at the targeted locus. In this study, we expanded epigenome editing to mouse primary T cells, with a focus on the *Foxp3* locus to elucidate epigenetic regulation mechanisms, and to advance future clinical usage in immunotherapy.

In this study, we applied dCas9-TET1CD and dCas9-p300CD to the *Foxp3* CNS2 and promoter locus, respectively, and attempted to generate Foxp3 stability-enhanced iTregs. We succeeded in epigenome editing at both loci, and the histone acetylation at the promoter locus strongly activated Foxp3 expression, but the DNA demethylation at the CNS2 locus slightly affected for Foxp3 expression.

dCas9-TET1CD demethylated the CNS2 locus, but did not intensely stabilize Foxp3 expression under inflammatory conditions. Although a certain level of stabilization was achieved by co-transduction with gRNA #C2-1 or #C2-7, we could not observe a similar statistically significant stabilization effect in comparison with dCas9-TET1CD catalytic activity. We speculate that demethylation efficiency is not sufficient for stable Foxp3 expression, as seen in nTregs, because even nTregs lose Foxp3 expression under inflammatory conditions, and the CNS2 locus was methylated in a parallel way [46, 53, 54]. Endogenous epigenetic modifiers could have excluded dCas9-TET1CD targeted to the CNS2 locus in iTregs under inflammatory conditions. In addition, dCas9-TET1CD itself impedes interaction of the *Foxp3* CNS2 locus with other endogenous transcriptional factors. As dCas9 itself is reported to inhibit transcription, and it is utilized in CRISPR interference technology [55]. When comparing dCas9-TET1CD with its catalytic inactive mutant, the difference in the Foxp3 stabilization effect was smaller than the difference between #C2-1 and #C2-7. We presume that dCas9-TET1CD (or even dCas9-TET1CD catalytic inactive mutant itself) targeted by #C2-7 was not protected from DNMT1 or DNMT3a recruited by inflammatory signals. In fact, in iTregs, the CNS2 locus was not passively regulated, and TET1CD catalytic inactive mutant decreased Foxp3 expression as measured by MFI, indicating that dCas9-TET1CD inactive mutant had a negative effect on Foxp3 transcription.

Recent research reported that the dCas9-TET1CD system in combination with repeating peptide array SunTag technology or engineered gRNA technology, in which bacteriophage MS2 RNA elements are inserted, succeeded in upregulating gene expression via considerable targeted demethylation of some promoter loci [43, 56]. Another study proposed that modified dCas9, with its degradation controlled by a chemical compound, could prevent dCas9 fusion proteins from remaining at targeted loci [57]. Additionally, SaCas9, which is smaller than SpCas9, is reported to overcome the size problem [58, 59]. In the future study, we plan to use these modified dCas systems in order to improve demethylation efficiency.

In accordance with a previous report, we confirmed that dCas9-p300CD could induce gene expression through histone acetylation. Like Hilton et al. [7], we observed that a single gRNA sequence is sufficient for transcriptional activation, and that this sequence is located approximately 60-bp upstream from the transcriptional start site. Additionally, we investigated the potential effects of dCas9-p300CD from two points of view. First, we clarified that artificial histone acetylation at the *Foxp3* promoter locus activated Foxp3 transcription, which could be enforced by the TGF-β signal. TGF-β signal is shown to accelerate Foxp3 induction by modifying the CNS1 enhancer locus [60]. It means that dCas9-p300CD targeted to the promoter locus did not mask the other enhancer locus function; rather, it could be activated. To dissect the locus specific regulation clearly, examining the activity of dCas9-p300CD in CNS1 locus-deficient cells is needed in the next study, since TGF-β signal was also reported to effect promoter locus [61, 62]. In addition to enhanced effectiveness of dCas9-p300CD by TGF-β signal, dCas9-p300CD targeted to the promoter locus was not interfered by inflammatory cytokines. The cytokine signals IL-12/STAT4, IL-4/STAT6, and IL-6/STAT3 did not mainly target the *Foxp3* promoter locus for downregulating Foxp3 transcription. Putative STAT6-binding sites even in *Foxp3* promoter locus are located further upstream of gRNA-targeted regions [63]. We presumed that dCas9-p300CD was salvaged from inactivation of the *Foxp3* gene locus by remaining at the *Foxp3* promoter locus under inflammatory conditions. It was expected that applying this system to various gene loci could identify a novel enhancer element and its regulatory stimuli or factors. Second, we showed that artificial histone acetylation not only sustained Foxp3 expression, but also reinforced Treg function. iTregs transduced with dCas9-p300CD and appropriate gRNA #P-4 highly expressed the Treg signature genes CD25 and CTLA-4, resulting in higher suppression activity. This indicates that dCas9-p300CD induced sufficient protein expression for engineering cellular functions.

Considering that dCas9-p300CD-mediated gene activation is observed only in a certain fraction, but not all of the transduced cells, effectiveness of dCas9-p300CD depends on each transduced cell. Examination of original chromatin states or accessibility of epigenetic modifier to the target locus in individual cells will clarify the more effective usage of epigenome editing. For example, H3K27me3, inactive epigenetic modification, is marked at the *Foxp3* promoter locus in conventional T cells [64]. Supposing that dCas9-p300CD has to rewrite this inactive mark with eraser help for transactivation, it is easy to speculate that effectiveness is decreased in such cells than H3K27 unmodified cells. Furthermore, memorization and stabilization of artificially induced epigenetic modification become issue. Our result suggested gene activation is strongly maintained in some cases. Whether this phenomenon was the results of epigenome editing or stable existence of epigenetic modifier is carefully examined in the next study.

Since *Foxp3* locus-targeted epigenome editing worked well in primary T cells for increasing Treg properties to some extent, further epigenetic modifications to other Treg-characteristic gene loci feasibly convert iTregs to nTregs. In fact, Foxp3 alone is not strictly and not sufficient to determine Treg signature, and multiple co-transcription factors have redundant functions for Treg physiology [65]. Primarily, we have to identify these gene loci and modify the epigenetic status in conjunction with the *Foxp3* locus by multiple gRNAs combination. Moreover, for future clinical usage of these epigenome-edited iTregs, optimization for therapeutic effect is required for functional Tregs. Expanding the target genes, suppressive cytokines, or inhibitory molecules seems to be effective for clinical usage. Other transcriptional activation systems can be applied for this purpose [66, 67].

Finally, we could not verify the function of epigenome-edited iTregs in the in vivo mouse model, because the transduction efficiency was not high enough to obtain a sufficient number of iTregs for disease model study. However, it has been reported that, in contrast to mice T cells, human T cells could be expanded using a rapid expansion protocol [68]. Moreover, lentivirus-mediated gene delivery methods have been established for clinical uses [69]. In our future study, we aim to apply our system to the human genome and human T cells, and expect its usage in medicine in the future.

## Conclusions

We proposed that applying epigenome editing to genes of interest would clarify gene expression regulation mechanisms. Our study firstly investigated the cross-talk of CRISPR-dCas9-based epigenome editing and endogenous cellular signaling in mouse primary T cells, focusing on *Foxp3* gene locus. dCas9-TET1CD and dCas9-p300CD edited specific CpG sites and chromatin histone, and we showed that subsequent gene activation was occurred by cooperating with the TGF-β signal (in the case of dCas9-p300CD) and was interfered by inflammatory cytokine signals (in the case of dCas9-TET1CD). It indicated that different epigenetic states at the *Foxp3* locus among cell types and cell culture conditions determined the effectiveness of dCas9-based epigenome editing. We expected that broad application to key genes for cell differentiation or human diseases would clarify epigenetic regulation mechanisms. We concluded that epigenome editing endowed innovation of clinical research and promise to clinical application in the next study.

## Additional files

**Additional file 1.** Amino acid sequences of dCas9-TET1CD and dCas9-p300CD.

**Additional file 2: Table S1.** Off-target analysis of selected gRNAs.

**Additional file 3: Figure S1.** Experimental scheme of Foxp3 stability assay. Upper, naïve CD4+ T cells (CD4+CD62L+hCD2-) were MACS sorted and cultured under iTreg skewing conditions, and on day 2, dCas9-fusion protein and gRNA were transduced with polybrene. The next day, iTregs were harvested and further cultured under iTreg or inflammatory cytokine conditions for 2 days. Foxp3 expression (hCD2) was analyzed by flow cytometry. Lower, representative Foxp3 expression. Flow cytometry plots show expression of Foxp3 (endogenous) and Foxp3(hCD2, surface indicator) in primary T cells from Foxp3-hCD52-hCD2 KI mice. **Figure S2**. dCas9-TET1CD-mediated Foxp3 stabilization. (A) Histogram of Foxp3(hCD2) in dCas9-TET1CD (GFP/DsRed(+/-)) and dCas9-TET1CD with #C2-7 (GFP/DsRed(+/+)) cells under inflammatory conditions. Related to Figure  3b. (B) Foxp3 mRNA expression same as in Figure 3b. Data are pooled from three independent experiments and represent the means ± SDs. **Figure S3**. TGF-β signal enhanced effectiveness of dCas9-p300CD-mediated Foxp3 induction. Foxp3 expression induced by low-dose TGF-β in the presence of LY2157299 or anti-TGF-β was monitored by Foxp3(hCD2) MFI. **Figure S4**. dCas9-p300CD and gRNA co-transduced iTregs. (A) Sorting strategy and purification. (B) Suppression assay of iTregs comparing dCas9-p300CD and #P-4 with dCas9-p300CD catalytic mutant.

## Abbreviations

Foxp3: Forkhead box P3
TGF-β: transforming growth factor beta
Tregs: regulatory T cells
TET: Ten-eleven translocation dioxygenase
DNMT: DNA methyltransferase
CNS: conserved non-coding DNA sequences
STAT: signal transducer and activator of transcription
CTLA-4: cytotoxic T-lymphocyte associated protein 4
