# Supplementary material for: Stabilization of Foxp3 expression by CRISPR-dCas9-based epigenome editing in mouse primary T cells
Source: Epigenetics Chromatin. 2017 May 8;10:24. doi: 10.1186/s13072-017-0129-1 (PMC5422987; doi:10.1186/s13072-017-0129-1)
Supplement: Supplementary file 1 — Additional file 1. Amino acid sequences of dCas9-TET1CD and dCas9-p300CD. [file 13072_2017_129_MOESM1_ESM.pdf]

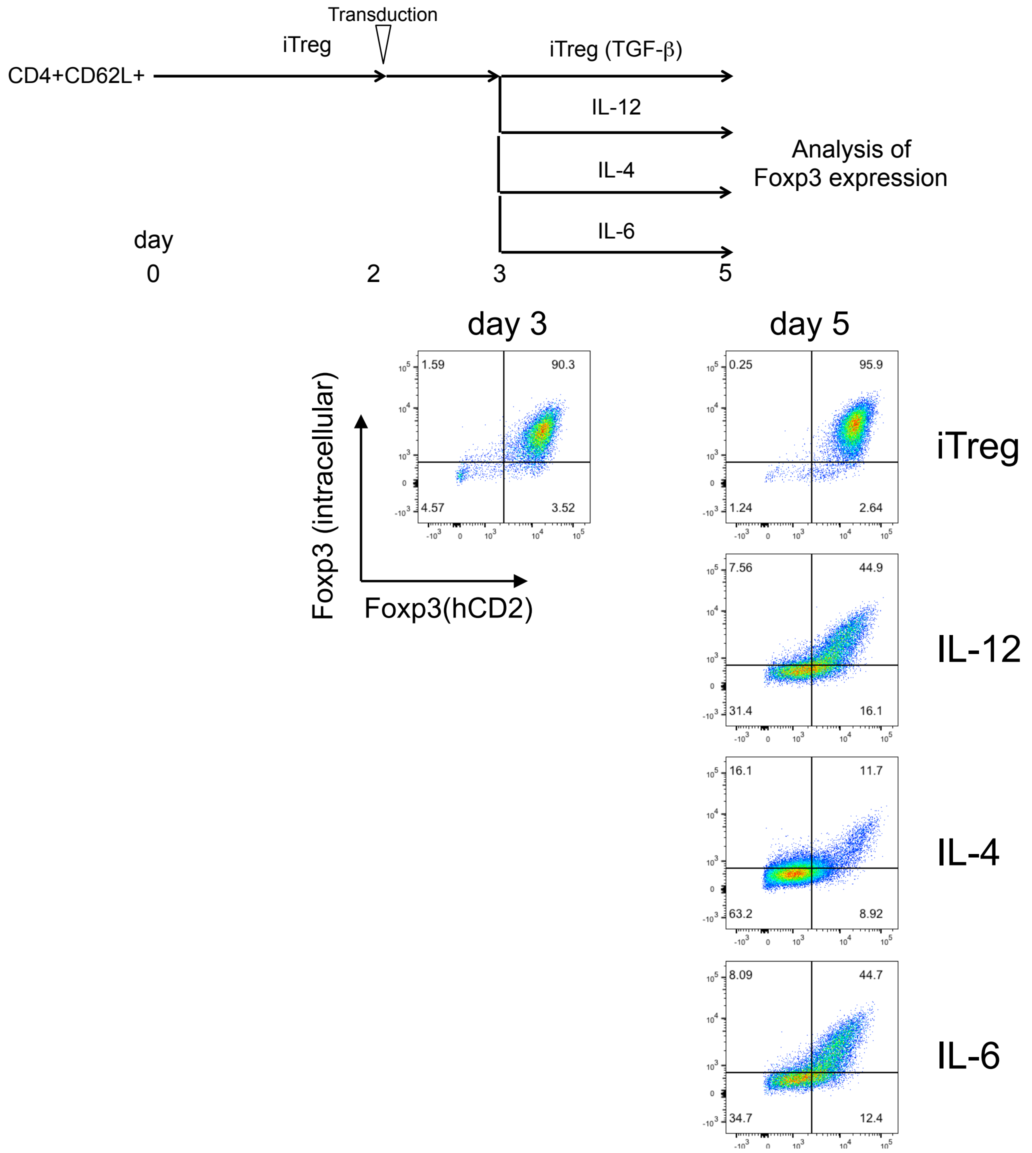

Supplementary Figure 1.
Okada et al.

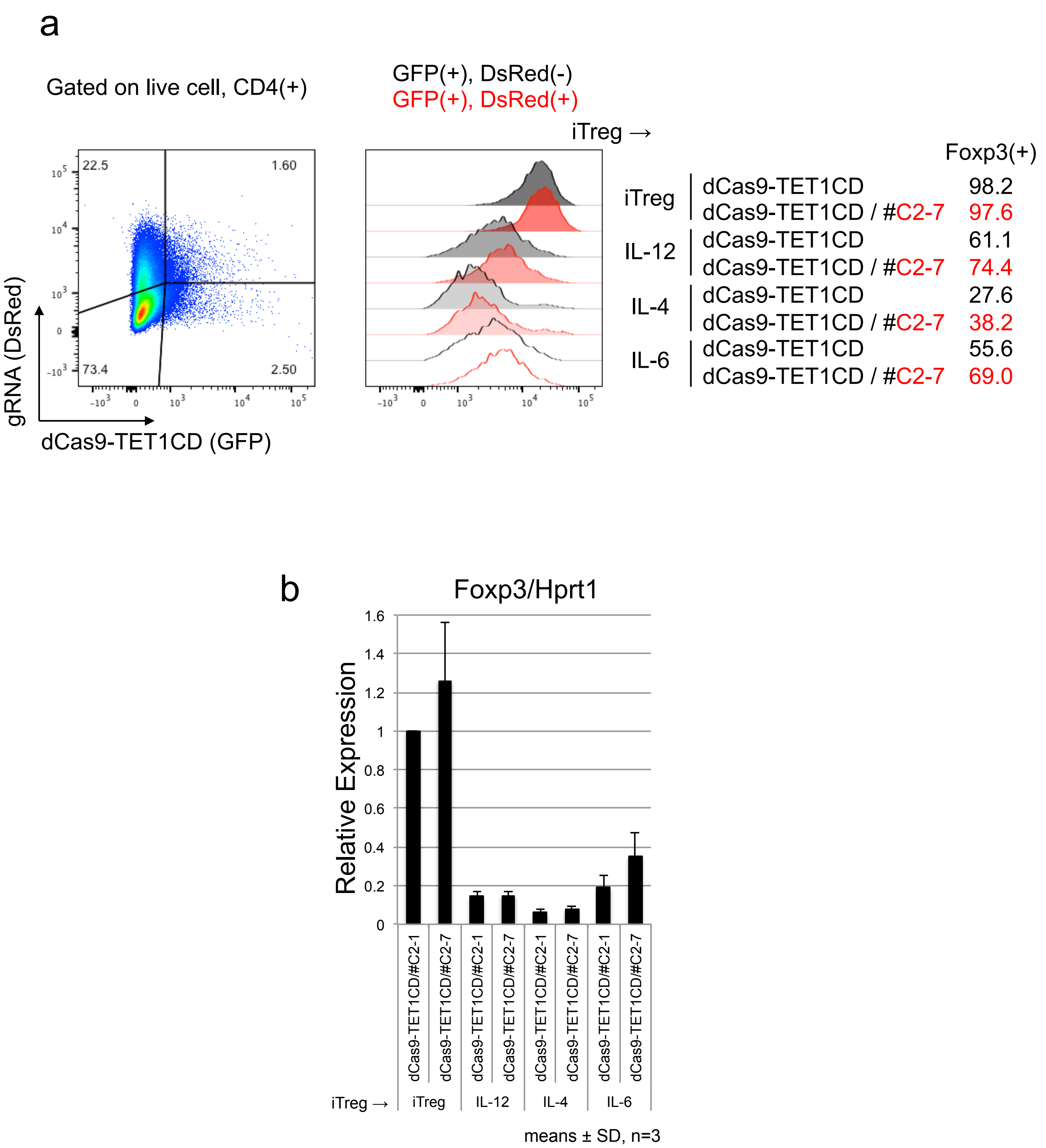

Supplementary Figure 2.
Okada et al.

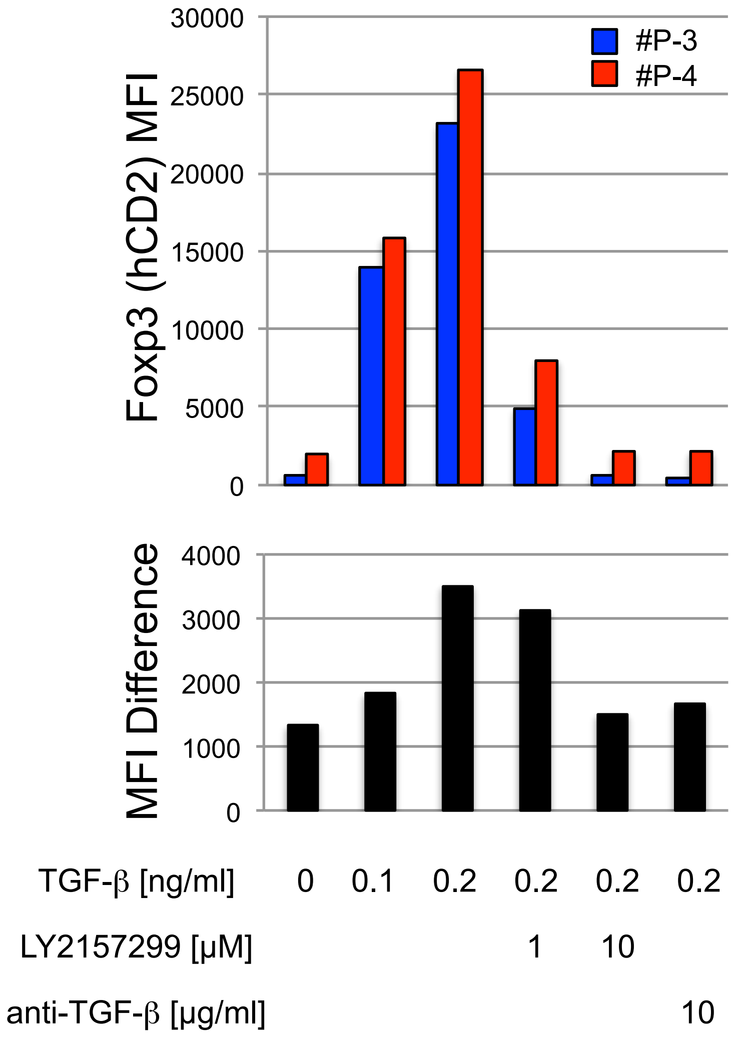

Supplementary Figure 3.
Okada et al.

a

Gated on live cell, CD4(+), hCD2(+)

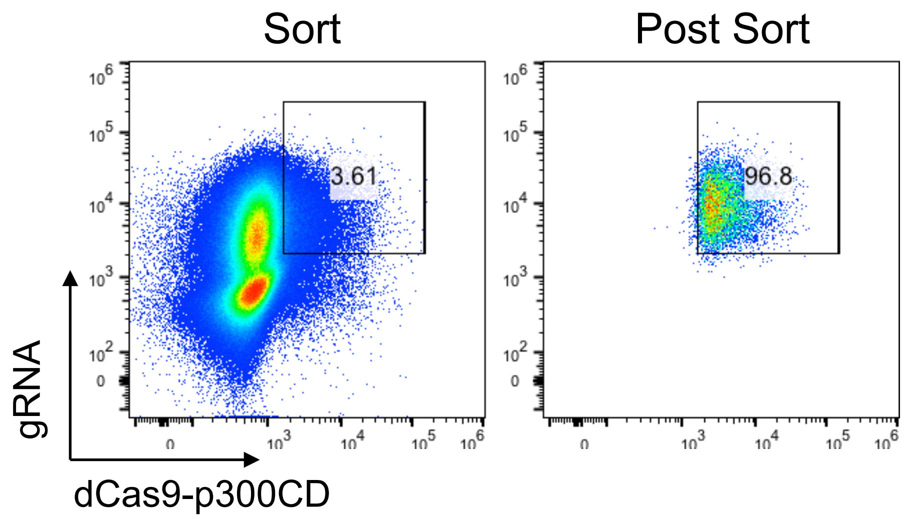

b

Gated on live cell, CD4(+)

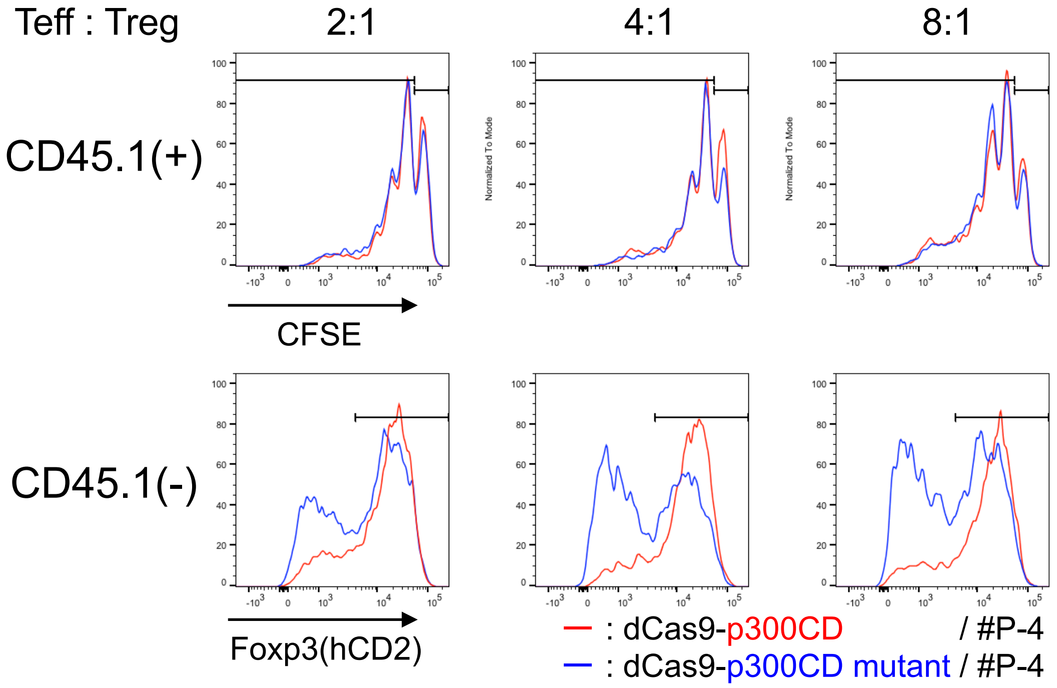

Supplementary Figure 4.
Okada et al.
