# Supplementary material for: Stabilization of Foxp3 expression by CRISPR-dCas9-based epigenome editing in mouse primary T cells
Source: Epigenetics Chromatin. 2017 May 8;10:24. doi: 10.1186/s13072-017-0129-1 (PMC5422987; doi:10.1186/s13072-017-0129-1)
Supplement: Supplementary file 2 — Additional file 2: Table S1. Off-target analysis of selected gRNAs. [file 13072_2017_129_MOESM2_ESM.pdf]

#C2-1
Species: Mouse (Mus musculus GRCm38/mm10)
Input: CTTGAGATTCTAAAATCCGTTGG
PAM: NGG

| Chromosome | start | end | strand | MM | target_seq | PAM | distance | position | gene name | gene id |
|---|---|---|---|---|---|---|---|---|---|---|
| chrX | 7583783 | 7583805 | + | 0 | CTTGAGATTCTAAAATCCGT | TGG | 1766 | Intronic | Foxp3 | ENSMUSG00000039521 |
| chr11 | 3980411 | 3980433 | − | 4 | TTTGAATTTGTAAAATCCGT | GGG | 407 | Intergenic | Pes1 | ENSMUSG00000020430 |
| chr15 | 10132210 | 10132232 | − | 3 | TTGGAGATTCTAACATCCGT | AGG | 45006 | Intergenic | Prlr | ENSMUSG00000005268 |
| chr8 | 29372301 | 29372323 | − | 4 | CTAGAGAAGCTAGAATCCGT | GGG | NA | Intergenic | NA | NA |
| chr7 | 113249882 | 113249904 | + | 4 | TTCTAGATTCTAAAAGCCGT | GGG | 15186 | Intronic | Arntl | ENSMUSG00000055116 |
| chr4 | 56147125 | 56147147 | − | 3 | CTTGACATTCAAAAAACCGT | GGG | 15793 | Intergenic | Gm12520 | ENSMUSG00000081290 |
| chr2 | 91091074 | 91091096 | − | 4 | CTTGAAACTCTTAGATCCGT | GGG | 764 | Intronic | Spi1 | ENSMUSG00000002111 |
| chr2 | 4019195 | 4019217 | − | 4 | CTTAACATTGTAAAAACCGT | AGG | 0 | Exonic | Frmd4a | ENSMUSG00000026657 |
| chr17 | 45157715 | 45157737 | − | 4 | CCAGAAATTCTAAAATCTGT | TGG | 38425 | Intergenic | Supt3 | ENSMUSG00000038954 |
| chr9 | 94689971 | 94689993 | − | 4 | CCTGAGAAGCTAAAATTCGT | TGG | 4714 | Intronic | Slc9a9 | ENSMUSG00000031129 |
| chr8 | 32059491 | 32059513 | + | 4 | CTTGGATTTCTAAAATCAGT | TGG | 43732 | Intergenic | Gm26578 | ENSMUSG00000097928 |
| chr2 | 124025759 | 124025781 | − | 4 | CTTCAGCTCCTAAAATCTGT | GGG | 64188 | Intergenic | Sema6d | ENSMUSG00000027200 |
| chr7 | 82799580 | 82799602 | − | 4 | CTTGGCATTTTAAAATCTGT | AGG | 21728 | Intergenic | Eftud1 | ENSMUSG00000038563 |
| chr17 | 19081540 | 19081562 | + | 4 | ATTTAGTTTCTAAAATCCAT | TGG | 229 | Intergenic | Vmn2r98 | ENSMUSG00000096717 |
| chr4 | 67727179 | 67727201 | + | 4 | TCTGAGATTCAAAAATCCTT | TGG | 93950 | Intergenic | AL589845.1 | ENSMUSG00000106523 |
| chr1 | 68213621 | 68213643 | + | 4 | CTTGACTTTCTGAAATCCCT | GGG | 14594 | Intronic | Erbb4 | ENSMUSG00000062209 |
| chr9 | 4762104 | 4762126 | − | 4 | CTGGAGAGTCTAAATTCTGT | TGG | 31684 | Intronic | Gria4 | ENSMUSG00000025892 |
| chrY | 44792736 | 44792758 | + | 4 | CTAGAGGTTCTAAGATCCCT | TGG | 25652 | Intergenic | Gm28887 | ENSMUSG00000102116 |
| chrY | 39189676 | 39189698 | − | 4 | CTAGAGGTTCTAAGATCCCT | TGG | 27596 | Intergenic | Gm29497 | ENSMUSG00000100588 |
| chrY | 45197825 | 45197847 | + | 4 | CTAGAGGTTCTAAGATCCCT | TGG | 27893 | Intergenic | Gm28457 | ENSMUSG00000099790 |
| chrY | 89363560 | 89363582 | + | 4 | CTAGAGGTTCTAAGATCCCT | TGG | 27946 | Intergenic | Gm28670 | ENSMUSG00000099982 |
| chrY | 45420944 | 45420966 | + | 4 | CTAGAGGTTCTAAGATCCCT | TGG | 27994 | Intergenic | Gm28458 | ENSMUSG00000100011 |
| chrY | 45015985 | 45016007 | + | 4 | CTAGAGGTTCTAAGATCCCT | TGG | 27995 | Intergenic | Gm29445 | ENSMUSG00000101802 |
| chrY | 43482379 | 43482401 | − | 4 | CTAGAGGTTCTAAGATCCCT | TGG | 28012 | Intergenic | Gm28568 | ENSMUSG00000100051 |
| chrY | 14835782 | 14835804 | + | 4 | CTAGAGGTTCTAAGATCCCT | TGG | 28036 | Intergenic | Gm37654 | ENSMUSG00000102251 |
| chrY | 89966247 | 89966269 | − | 4 | CTAGAGGTTCTAAGATCCCT | TGG | 31234 | Intergenic | Gm28930 | ENSMUSG00000102011 |
| chrY | 19548385 | 19548407 | + | 4 | CTAGAGGTTCTAAGATCCCT | TGG | 33371 | Intergenic | Gm21763 | ENSMUSG00000102552 |
| chrY | 40032667 | 40032689 | + | 4 | CTAGAGGTTCTAAGATCCCT | TGG | 43686 | Intergenic | Gm28371 | ENSMUSG00000101124 |
| chrX | 47938083 | 47938105 | − | 4 | CAAGAGATTCTAAAATACCT | GGG | 194 | Intronic | Ocrl | ENSMUSG00000001173 |
| chr2 | 51177376 | 51177398 | − | 4 | CTCTAGATTCTAAAATTCAT | TGG | 28265 | Intergenic | Rnd3 | ENSMUSG00000017144 |
| chr1 | 143694111 | 143694133 | − | 4 | CTTGAAACTCTAAAATCTCT | AGG | 1260 | Intronic | Cdc73 | ENSMUSG00000026361 |
| chr1 | 4621544 | 4621566 | − | 4 | CTTTAGAGTCTAAAATCAGC | AGG | 10138 | Intergenic | Gm7369 | ENSMUSG00000102735 |
| chr15 | 74380095 | 74380117 | + | 4 | GTTGTGATTCTAAAATCCAA | GGG | NA | Intergenic | NA | NA |
| chr11 | 54903827 | 54903849 | + | 4 | TTTGAGTTTCTAAAATCCTC | AGG | 775 | Intronic | Gpx3 | ENSMUSG00000018339 |
| chr7 | 43460910 | 43460932 | − | 4 | CTTGACCTTCTAAAATCCCA | TGG | 2205 | Intronic | RP23-74K24.2 | ENSMUSG00000107482 |
| chr19 | 38087782 | 38087804 | − | 4 | CTTGAATTTCTAAAATCCAA | GGG | 9275 | Intergenic | Ffar4 | ENSMUSG00000054200 |
| chr1 | 83742117 | 83742139 | + | 4 | CTTGAAACTCTAAAATCCCA | AGG | 37452 | Intronic | 4933436I20Rik | ENSMUSG00000100580 |

#C2-7
Species: Mouse (Mus musculus GRCm38/mm10)
Input: TCCGGCCGCCATGACGTCAATGG
PAM: NGG

| Chromosome | start | end | strand | MM | target_seq | PAM | distance | position | gene name | gene id |
|---|---|---|---|---|---|---|---|---|---|---|
| chrX | 7584134 | 7584156 | + | 0 | TCCGGCCGCCATGACGTCAA | TGG | 1415 | Intronic | Foxp3 | ENSMUSG00000039521 |
| chr2 | 134688521 | 134688543 | − | 4 | TGCAGCAGCCAGGACGTCAA | AGG | 42252 | Intergenic | Gm14037 | ENSMUSG00000081243 |
| chr7 | 100031801 | 100031823 | + | 4 | TCATGGCGCCATGGCGTCAA | GGG | 1821 | Intronic | Chrdl2 | ENSMUSG00000030732 |
| chr7 | 123423065 | 123423087 | + | 4 | TGCTGCTGCCATGACTTCAA | GGG | 267 | Intronic | Lcmt1 | ENSMUSG00000030763 |
| chr8 | 106855013 | 106855035 | + | 4 | TGCAGCGGCCATGACCTCAA | GGG | 3574 | Intergenic | Tango6 | ENSMUSG00000041949 |
| chr17 | 44413180 | 44413202 | − | 4 | TCCTGCCATCATGACGACAA | TGG | 82785 | Intergenic | Runx2 | ENSMUSG00000039153 |
| chr6 | 107511434 | 107511456 | + | 4 | TCCAGCTGCCATGTCCTCAA | TGG | 6978 | Intergenic | RP23-331P17. | ENSMUSG00000107674 |
| chr9 | 43699223 | 43699245 | + | 4 | ACCTGCCGCCATGACAGCAA | GGG | 45331 | Intergenic | Pvrl1 | ENSMUSG00000032012 |
| chr3 | 108562544 | 108562566 | − | 4 | TCCGGTTGCCATGACCACAA | AGG | 78 | Intergenic | Tmem167b | ENSMUSG00000068732 |
| chr2 | 36136631 | 36136653 | − | 4 | TGCGGGCGTCATGACGTCAC | GGG | 0 | Exonic | Mrrf | ENSMUSG00000026887 |
| chr8 | 110024210 | 110024232 | − | 4 | TCAGGCAGCCATGACCTAAA | AGG | 4843 | Intergenic | Chst4 | ENSMUSG00000035930 |
| chr5 | 138766621 | 138766643 | + | 4 | TCCTGCAGCCATGAAGTCAG | GGG | 0 | Exonic | Fam20c | ENSMUSG00000025854 |
| chr9 | 35040901 | 35040923 | + | 4 | TCCGGAAGCCATGACTTCAC | TGG | 4185 | Intergenic | Kirrel3 | ENSMUSG00000032036 |

#P-3
Species: Mouse (Mus musculus GRCm38/mm10)
Input: CACACTCATCAAAAAAAAATTGG
PAM: NGG

| Chromosome | start | end | strand | MM | target_seq | PAM | distance | position | gene name | gene id |
|---|---|---|---|---|---|---|---|---|---|---|
| chrX | 7579569 | 7579591 | + | 0 | CACACTCATCAAAAAAAAAT | TGG | 0 | Exonic | Ppp1r3fos | ENSMUSG00000039545 |
| chr4 | 132088926 | 132088948 | + | 3 | CCCTGTCATCAAAAAAAAAT | AGG | 4943 | Intergenic | Gm13214 | ENSMUSG00000083368 |
| chrX | 52471288 | 52471310 | + | 4 | GTGAGTCATCAAAAAAAAAT | GGG | 42154 | Intronic | Gpc3 | ENSMUSG00000055653 |
| chr15 | 11692320 | 11692342 | − | 4 | TATGTTCATCAAAAAAAAAT | GGG | NA | Intergenic | NA | NA |
| chrX | 51433475 | 51433497 | − | 4 | CCTGCCCATCAAAAAAAAAT | GGG | 4731 | Intronic | Hs6st2 | ENSMUSG00000062184 |
| chrX | 141256352 | 141256374 | + | 4 | AACTCAAATCAAAAAAAAAT | GGG | 22144 | Intronic | Col4a6 | ENSMUSG00000031273 |
| chr9 | 7996895 | 7996917 | − | 3 | CACTCTGCTCAAAAAAAAAT | AGG | 0 | Exonic | Yap1 | ENSMUSG00000053110 |
| chr15 | 60018290 | 60018312 | + | 4 | CAACCCAATCAAAAAAAAAT | GGG | 1672 | Intergenic | 4933412E24Rik | ENSMUSG00000071749 |
| chr1 | 5042638 | 5042660 | − | 4 | CAACCCAATCAAAAAAAAAT | GGG | 20119 | Intronic | Rgs20 | ENSMUSG00000002459 |
| chr18 | 51408393 | 51408415 | − | 4 | CAACCCAATCAAAAAAAAAT | GGG | NA | Intergenic | NA | NA |
| chr14 | 75255177 | 75255199 | − | 3 | TACACACATTAAAAAAAAAT | AGG | 757 | Intronic | Cpb2 | ENSMUSG00000021999 |
| chr9 | 109485579 | 109485601 | − | 3 | CACTCACATAAAAAAAAAAT | GGG | 271 | Intronic | Fbxw19 | ENSMUSG00000074061 |
| chr1 | 111034384 | 111034406 | − | 4 | CAATATCAGCAAAAAAAAAT | TGG | 56800 | Intergenic | Cdh19 | ENSMUSG00000047216 |
| chr7 | 26146453 | 26146475 | − | 4 | TAGAATCATAAAAAAAAAAT | AGG | 1673 | Intronic | RP23-174D7.4 | ENSMUSG00000108544 |
| chrX | 78703443 | 78703465 | − | 4 | AAAAATCATAAAAAAAAAAT | CGG | 20255 | Intergenic | Gm14747 | ENSMUSG00000085610 |

| | | | | | | | | | | | |
|---|---|---|---|---|---|---|---|---|---|---|---|
| chr13 | 61731925 | 61731947 | + | 4 | GATATTCATAAAAAAAAAT | TGG | | 67954 | Intergenic | Gm19961 | ENSMUSG00000095905 |
| chr2 | 102691568 | 102691590 | − | 3 | CAGAGTCATCTAAAAAAAAT | AGG | | 2375 | Intronic | Slc1a2 | ENSMUSG00000005089 |
| chr11 | 99604483 | 99604505 | − | 4 | AACAAGCAACAAAAAAAAAT | AGG | | 1175 | Intergenic | Gm11938 | ENSMUSG00000057674 |
| chr5 | 43659389 | 43659411 | + | 4 | AACAAACAACAAAAAAAAAT | AGG | | 2935 | Intergenic | Cc2d2a | ENSMUSG00000039765 |
| chrX | 61096487 | 61096509 | − | 4 | CTAATTCATAAAAAAAAAAT | GGG | | 4753 | Intergenic | Gm14663 | ENSMUSG00000083543 |
| chr6 | 144760427 | 144760449 | − | 4 | AACTTTCATTAAAAAAAAAT | TGG | | 544 | Intronic | Sox5os4 | ENSMUSG00000086282 |
| chr15 | 20348716 | 20348738 | + | 4 | TAAACCCATTAAAAAAAAAT | GGG | NA | | Intergenic | NA | NA |
| chr10 | 75477656 | 75477678 | − | 4 | CAAAAACAACAAAAAAAAAT | TGG | | 28605 | Intronic | Gucd1 | ENSMUSG00000033416 |
| chr15 | 25026774 | 25026796 | + | 4 | TCCACTTATAAAAAAAAAAT | CGG | | 430 | Intronic | Gm2824 | ENSMUSG00000097452 |
| chr16 | 25167179 | 25167201 | − | 4 | CAGGGTCATAAAAAAAAAAT | AGG | | 98121 | Intergenic | A230028O05Ri | ENSMUSG00000096960 |
| chr2 | 69687728 | 69687750 | + | 4 | CCCTCCCATTAAAAAAAAAT | GGG | | 588 | Intronic | Fastkd1 | ENSMUSG00000027086 |
| chr14 | 74623048 | 74623070 | − | 3 | CAGACTTATCTAAAAAAAAT | GGG | | 17770 | Intergenic | Htr2a | ENSMUSG00000034997 |
| chr1 | 149567938 | 149567960 | − | 4 | CAAAATAAACAAAAAAAAAT | GGG | NA | | Intergenic | NA | NA |
| chr1 | 177208323 | 177208345 | + | 4 | GACTCTAATGAAAAAAAAAT | AGG | | 21546 | Intronic | Gm37463 | ENSMUSG00000102427 |
| chr19 | 4969684 | 4969706 | + | 4 | CTCAAACATTAAAAAAAAAT | AGG | | 2689 | Intergenic | Mrpl11 | ENSMUSG00000024902 |
| chr12 | 41949616 | 41949638 | − | 3 | CAGATTCATCACAAAAAAAT | GGG | | 5343 | Intronic | Immp2l | ENSMUSG00000056899 |
| chr8 | 60184386 | 60184408 | + | 4 | CTCTCTAATGAAAAAAAAAT | GGG | NA | | Intergenic | NA | NA |
| chr18 | 23494666 | 23494688 | + | 4 | AAGACTCCTGAAAAAAAAAT | GGG | | 19424 | Intronic | Dtna | ENSMUSG00000024302 |
| chr14 | 20540781 | 20540803 | − | 3 | CACACCCTTAAAAAAAAAAT | CGG | | 5276 | Intronic | 1810062O18Ril | ENSMUSG00000084925 |
| chrY | 52373157 | 52373179 | + | 4 | CATTCTGATAAAAAAAAAAT | GGG | | 10820 | Intergenic | Gm28738 | ENSMUSG00000099565 |
| chrY | 64969870 | 64969892 | + | 4 | CATTCTGATAAAAAAAAAAT | GGG | | 10822 | Intergenic | Gm28255 | ENSMUSG00000099386 |
| chrY | 72148140 | 72148162 | + | 4 | CATTCTGATAAAAAAAAAAT | GGG | | 10829 | Intergenic | Gm29181 | ENSMUSG00000099731 |
| chrY | 49670071 | 49670093 | − | 4 | CATTCTGATAAAAAAAAAAT | GGG | | 10838 | Intergenic | Gm28682 | ENSMUSG00000100564 |
| chrY | 62289786 | 62289808 | − | 4 | CATTCTGATAAAAAAAAAAT | GGG | | 10842 | Intergenic | Gm28185 | ENSMUSG00000099626 |
| chrX | 132699573 | 132699595 | + | 4 | CAACCTAATTAAAAAAAAAT | GGG | | 17484 | Intergenic | Gm22143 | ENSMUSG00000088026 |
| chr10 | 113684543 | 113684565 | − | 4 | CATTCTAATAAAAAAAAAAT | GGG | NA | | Intergenic | NA | NA |
| chr1 | 145080821 | 145080843 | + | 4 | CCAACTCTTAAAAAAAAAAT | AGG | | 84108 | Intergenic | Gm6550 | ENSMUSG00000099617 |
| chr18 | 33285595 | 33285617 | − | 4 | CACCTACATTAAAAAAAAAT | TGG | | 71733 | Intergenic | Stard4 | ENSMUSG00000024378 |
| chr1 | 142266084 | 142266106 | + | 4 | CACTGCCATGAAAAAAAAAT | TGG | NA | | Intergenic | NA | NA |
| chr1 | 74642970 | 74642992 | − | 4 | AATACTGATCTAAAAAAAAT | TGG | | 6076 | Intergenic | Stk36 | ENSMUSG00000033276 |
| chr5 | 59791361 | 59791383 | + | 4 | AACACAAATGAAAAAAAAAT | AGG | | 13038 | Intergenic | Gm43043 | ENSMUSG00000104889 |
| chr6 | 5450315 | 5450337 | + | 4 | GACACAGATGAAAAAAAAAT | AGG | | 17293 | Intergenic | Asb4 | ENSMUSG00000042607 |
| chr2 | 173621421 | 173621443 | + | 4 | CATAATGATGAAAAAAAAAT | TGG | | 4480 | Intronic | Ppp4r1l-ps | ENSMUSG00000055897 |
| chr14 | 66861086 | 66861108 | + | 4 | TTCACTCAGAAAAAAAAAAT | GGG | | 1365 | Intronic | Dpysl2 | ENSMUSG00000022048 |
| chr10 | 32601465 | 32601487 | − | 4 | AGCACTCACAAAAAAAAAAT | GGG | NA | | Intronic | NA | NA |
| chr11 | 4965261 | 4965283 | + | 4 | CTCCCTCCTTAAAAAAAAAT | TGG | | 17197 | Intergenic | Nefh | ENSMUSG00000020396 |
| chr6 | 106476791 | 106476813 | + | 4 | GACACTTTACAAAAAAAAAT | AGG | | 12780 | Intronic | Cntn4 | ENSMUSG00000064293 |
| chr5 | 124550378 | 124550400 | − | 3 | CACACACAAAAAAAAAAAAT | TGG | | 0 | Exonic | Tmed2 | ENSMUSG00000029390 |
| chr13 | 117032245 | 117032267 | + | 3 | CACACCCAAAAAAAAAAAAT | AGG | | 6729 | Intergenic | Parp8 | ENSMUSG00000021725 |
| chr16 | 46812755 | 46812777 | − | 4 | AACTCTCAGAAAAAAAAAAT | GGG | NA | | Intergenic | NA | NA |
| chr17 | 51001564 | 51001586 | − | 4 | CATAATCTTAAAAAAAAAAT | TGG | | 16890 | Intronic | Tbc1d5 | ENSMUSG00000023923 |
| chr7 | 59150755 | 59150777 | − | 3 | CACACTGAATAAAAAAAAAT | GGG | | 77973 | Intergenic | Ube3a | ENSMUSG00000025326 |
| chrX | 79673766 | 79673788 | + | 4 | GGCACTGATCAGAAAAAAAT | AGG | | 2331 | Intergenic | Mageb16 | ENSMUSG00000046942 |
| chr3 | 143644226 | 143644248 | + | 4 | CATGCTCAGAAAAAAAAAAT | GGG | | 642 | Intergenic | Gm38006 | ENSMUSG00000103816 |
| chrX | 89826628 | 89826650 | − | 4 | CACAACAATAAAAAAAAAAT | TGG | | 20502 | Intergenic | Gm7108 | ENSMUSG00000083253 |
| chr15 | 38192561 | 38192583 | − | 4 | CACAGAAATTAAAAAAAAAT | GGG | | 26620 | Intergenic | Odf1 | ENSMUSG00000061923 |
| chr7 | 90875141 | 90875163 | + | 4 | CACAGCAATAAAAAAAAAAT | AGG | NA | | Intergenic | NA | NA |
| chr17 | 4370422 | 4370444 | + | 4 | CCCAATCAAAAAAAAAAAAT | GGG | NA | | Intergenic | NA | NA |
| chrX | 81933584 | 81933606 | + | 4 | AAAACTAATCAGAAAAAAAT | GGG | NA | | Intergenic | NA | NA |
| chr3 | 52621792 | 52621814 | + | 4 | GACACACAAAAAAAAAAAAT | AGG | | 7956 | Intergenic | Gm10293 | ENSMUSG00000070490 |
| chr10 | 56531248 | 56531270 | − | 4 | TACACACACAAAAAAAAAAT | GGG | NA | | Intergenic | NA | NA |
| chr6 | 63776611 | 63776633 | + | 4 | CAAAATCAGAAAAAAAAAAT | GGG | | 7219 | Intronic | RP23-133M12. | ENSMUSG00000108068 |
| chr11 | 120080058 | 120080080 | + | 4 | CATACTTTTTAAAAAAAAAT | AGG | | 1164 | Intronic | Cep131 | ENSMUSG00000039781 |
| chr4 | 109190294 | 109190316 | + | 4 | CATACTTTTTAAAAAAAAAT | TGG | | 11789 | Intronic | Osbpl9 | ENSMUSG00000028559 |
| chr2 | 93140629 | 93140651 | + | 4 | AACACCCCTCCAAAAAAAAT | AGG | | 42385 | Intergenic | Gm13802 | ENSMUSG00000087559 |
| chr11 | 92769292 | 92769314 | + | 4 | CACAACCTTTAAAAAAAAAT | GGG | | 717 | Intergenic | Gm11500 | ENSMUSG00000081363 |
| chr18 | 36677637 | 36677659 | + | 4 | CAAACCCAGAAAAAAAAAAT | AGG | | 132 | Intronic | Sra1 | ENSMUSG00000006050 |
| chr10 | 62656250 | 62656272 | + | 4 | AACACTTAAAAAAAAAAAAT | CGG | | 2771 | Intergenic | Stox1 | ENSMUSG00000036923 |
| chr6 | 38992400 | 38992422 | − | 4 | AACACTTAAAAAAAAAAAAT | GGG | | 8075 | Intronic | Gm42962 | ENSMUSG00000106737 |
| chr7 | 68940531 | 68940553 | − | 4 | TACACTAACAAAAAAAAAAT | TGG | | 11547 | Intergenic | Gm23795 | ENSMUSG00000065940 |
| chr3 | 11271385 | 11271407 | + | 4 | TACACTTAAAAAAAAAAAAT | TGG | NA | | Intergenic | NA | NA |
| chr1 | 174373723 | 174373745 | − | 3 | GACACTCTTCAATAAAAAAT | TGG | | 802 | Intergenic | Olfr416-ps1 | ENSMUSG00000102920 |
| chr10 | 125534311 | 125534333 | − | 4 | ACCACTCATTTAAAAAAAAT | GGG | NA | | Intergenic | NA | NA |
| chr8 | 61512044 | 61512066 | + | 4 | CTCACTTAAAAAAAAAAAAT | TGG | | 1180 | Intergenic | Palld | ENSMUSG00000058056 |
| chr2 | 11088655 | 11088677 | + | 4 | AAGACTCATTTAAAAAAAAT | GGG | | 742 | Intergenic | Gm26478 | ENSMUSG00000084560 |
| chrX | 95246470 | 95246492 | + | 4 | AAAACTCATACAAAAAAAAT | TGG | | 6424 | Intergenic | Gm14807 | ENSMUSG00000080298 |
| chr15 | 90696252 | 90696274 | + | 3 | CACACACATTGAAAAAAAAT | AGG | | 16820 | Intergenic | Cpne8 | ENSMUSG00000052560 |
| chr3 | 111694740 | 111694762 | − | 4 | CAAACTAACGAAAAAAAAAT | AGG | | 94730 | Intergenic | Gm42905 | ENSMUSG00000105882 |
| chrX | 156911201 | 156911223 | + | 4 | CATACTAAAGAAAAAAAAAT | GGG | NA | | Intergenic | NA | NA |
| chr13 | 60369049 | 60369071 | + | 4 | CACTCTTAGAAAAAAAAAAT | TGG | | 75277 | Intergenic | Gm24999 | ENSMUSG00000080668 |
| chr10 | 32685087 | 32685109 | + | 4 | CTCACTCGGAAAAAAAAAAT | AGG | NA | | Intronic | NA | NA |
| chrX | 150326938 | 150326960 | + | 4 | CACAGTAAAAAAAAAAAAAT | AGG | | 6033 | Intronic | Gm15104 | ENSMUSG00000087360 |
| chr13 | 56634027 | 56634049 | − | 4 | CAACCTCATTTAAAAAAAAT | TGG | | 1038 | Intronic | Tgfbi | ENSMUSG00000035493 |
| chr14 | 38183959 | 38183981 | + | 4 | CACACAAAAAAAAAAAAAAT | GGG | NA | | Intergenic | NA | NA |
| chr1 | 16001073 | 16001095 | − | 4 | CCCACACATGGAAAAAAAAT | TGG | | 52240 | Intergenic | Gm7634 | ENSMUSG00000100652 |
| chr16 | 77246059 | 77246081 | − | 4 | AACACTGATGTAAAAAAAAT | GGG | | 8809 | Intronic | Mir99ahg | ENSMUSG00000090386 |
| chr1 | 44403677 | 44403699 | − | 4 | AACACTAATACAAAAAAAAT | AGG | | 9230 | Intergenic | Gm28893 | ENSMUSG00000100769 |
| chrX | 42631005 | 42631027 | + | 4 | CACAAACATACAAAAAAAAT | AGG | | 11549 | Intronic | Tenm1 | ENSMUSG00000016150 |
| chr13 | 54547670 | 54547692 | + | 4 | CCCATACATCAAATAAAAAT | AGG | | 331 | Intronic | Simc1 | ENSMUSG00000043183 |
| chr6 | 75807033 | 75807055 | − | 4 | CTCACTACTCAATAAAAAAT | GGG | | 24277 | Intergenic | Gm9001 | ENSMUSG00000080824 |
| chr11 | 27801123 | 27801145 | − | 4 | AACAATAATCAAACAAAAAT | TGG | | 81967 | Intergenic | Gm12079 | ENSMUSG00000084064 |
| chr6 | 22061717 | 22061739 | + | 4 | CAGGCTCATCTCAAAAAAAT | AGG | | 1765 | Intronic | Cped1 | ENSMUSG00000062980 |
| chr16 | 59951780 | 59951802 | − | 4 | CACTCCCATAAGAAAAAAAT | GGG | | 2850 | Intronic | Epha6 | ENSMUSG00000055540 |
| chr15 | 42826576 | 42826598 | + | 4 | CCCACCCACCAACAAAAAAT | GGG | NA | | Intergenic | NA | NA |
| chr2 | 124841029 | 124841051 | + | 4 | CAAACTGATAAGAAAAAAAT | TGG | NA | | Intergenic | NA | NA |

| | | | | | | | | | | | |
|---|---|---|---|---|---|---|---|---|---|---|---|
| chr7 | 89381382 | 89381404 | − | 3 | CGCACTCATAAAACAAAAAT | AGG | | 12963 | Intronic | Gm28748 | ENSMUSG00000101924 |
| chr2 | 43854206 | 43854228 | + | 4 | GACACACATCTGAAAAAAAT | GGG | | 2578 | Intronic | Arhgap15 | ENSMUSG00000049744 |
| chr11 | 5217092 | 5217114 | + | 4 | CTCAATCATAAAGAAAAAAT | TGG | | 1561 | Intronic | Kremen1 | ENSMUSG00000020393 |
| chr11 | 16115910 | 16115932 | + | 4 | CACATTCCACAGAAAAAAAT | TGG | | 77482 | Intergenic | Gm37319 | ENSMUSG00000104323 |
| chrX | 41199611 | 41199633 | + | 4 | CACACACTTTGAAAAAAAAT | TGG | | 48942 | Intergenic | Gm5385 | ENSMUSG00000081880 |
| chr8 | 114216907 | 114216929 | + | 4 | GAGACTCATCCAGAAAAAAT | TGG | | 10960 | Intronic | Vat1l | ENSMUSG00000046844 |
| chr6 | 117224017 | 117224039 | + | 4 | CATACATATCAAATAAAAAT | AGG | | 9951 | Intergenic | Rpl28-ps4 | ENSMUSG00000090549 |
| chr1 | 171664882 | 171664904 | + | 4 | CACAAACAACAACAAAAAAT | CGG | | 11780 | Intergenic | A630035G10Ri | ENSMUSG00000103083 |
| chr4 | 126013592 | 126013614 | + | 4 | CACACACCACATAAAAAAAT | AGG | | 10936 | Intergenic | Csf3r | ENSMUSG00000028859 |
| chr1 | 158010035 | 158010057 | − | 4 | CACACTGTTGGAAAAAAAAT | AGG | | 47186 | Intronic | Gm38256 | ENSMUSG00000102789 |
| chr8 | 23775579 | 23775601 | − | 3 | CACACTGAACAAACAAAAAT | GGG | | 21741 | Intronic | Zmat4 | ENSMUSG00000037492 |
| chr10 | 37025826 | 37025848 | + | 4 | CATACACATGAAGAAAAAAT | TGG | | 23937 | Intergenic | Hdac2 | ENSMUSG00000019777 |
| chr8 | 117060110 | 117060132 | − | 3 | CTCACTCACCAAAATAAAAT | AGG | | 376 | Intronic | Pkd1l2 | ENSMUSG00000034416 |
| chr8 | 95816602 | 95816624 | − | 3 | CACACACATAAAATAAAAAT | GGG | | 156 | Intronic | 4930513N10Rik | ENSMUSG00000074136 |
| chr16 | 12296093 | 12296115 | − | 3 | CACACACATAAAATAAAAAT | AGG | | 25191 | Intergenic | Shisa9 | ENSMUSG00000022494 |
| chr4 | 142657335 | 142657357 | − | 4 | CCAACTGATCAAAAGAAAAT | GGG | NA | | Intergenic | NA | NA |
| chr3 | 147193983 | 147194005 | − | 4 | AACATTCATAAAACAAAAAT | AGG | | 1065 | Intergenic | Gm6074 | ENSMUSG00000103099 |
| chr7 | 46346426 | 46346448 | − | 4 | AAACACTAAACAAATAAAAT | GGG | | 3456 | Intergenic | Gm38176 | ENSMUSG00000103826 |
| chr6 | 126469680 | 126469702 | + | 4 | CACACACTGCAAGAAAAAAT | AGG | | 62849 | Intergenic | Kcna5 | ENSMUSG00000045534 |
| chr15 | 4570368 | 4570390 | − | 4 | TGCTCTCATCAAAAAGAAAT | AGG | | 4334 | Intronic | Plcxd3 | ENSMUSG00000049148 |
| chr18 | 45073915 | 45073937 | − | 4 | CATATTGATCAAAATAAAAT | GGG | | 15389 | Intergenic | Gm26928 | ENSMUSG00000098244 |
| chr6 | 35353495 | 35353517 | − | 4 | CCCCCTCTTCAAAACAAAAT | CGG | | 16670 | Intergenic | 1700065J11Rik | ENSMUSG00000107341 |
| chr6 | 42674597 | 42674619 | + | 3 | CACACACATCTAAGAAAAAT | TGG | | 423 | Intronic | Tcaf1 | ENSMUSG00000036667 |
| chr5 | 113084060 | 113084082 | − | 4 | CACCATGATCAAAATAAAAT | TGG | | 2241 | Intergenic | 2900026A02Rik | ENSMUSG00000051339 |
| chr9 | 67372263 | 67372285 | + | 4 | CACACGCCTCTTAAAAAAAT | TGG | | 1496 | Intronic | Tln2 | ENSMUSG00000052698 |
| chr10 | 40425081 | 40425103 | − | 4 | CACAGTCTTAAATAAAAAAT | GGG | | 9201 | Intronic | Cdk19 | ENSMUSG00000038481 |
| chr9 | 114703269 | 114703291 | + | 4 | CAAACACATAAAATAAAAAT | AGG | | 1763 | Intronic | Dync1li1 | ENSMUSG00000032435 |
| chr17 | 35599132 | 35599154 | − | 4 | CATACACATAAAATAAAAAT | AGG | | 2395 | Intergenic | Sfta2 | ENSMUSG00000090509 |
| chr11 | 95419490 | 95419512 | − | 4 | CATACACATAAAATAAAAAT | AGG | | 5086 | Intronic | Spop | ENSMUSG00000057522 |
| chr5 | 24276066 | 24276088 | + | 4 | CATACACATTAAATAAAAAT | AGG | | 19029 | Intergenic | Gm9836 | ENSMUSG00000081071 |
| chr14 | 60120497 | 60120519 | + | 4 | CATACACATAAAATAAAAAT | AGG | | 20544 | Intronic | Gm6913 | ENSMUSG00000075288 |
| chr9 | 30253775 | 30253797 | + | 4 | CATACACATAAAATAAAAAT | AGG | | 38071 | Intergenic | Gm26435 | ENSMUSG00000096018 |
| chr16 | 40465289 | 40465311 | + | 4 | CATACACATAAAATAAAAAT | AGG | | 97352 | Intronic | Gm27887 | ENSMUSG00000098472 |
| chr10 | 129812675 | 129812697 | + | 4 | CAAAGTCCTCAAAATAAAAT | AGG | | 10152 | Intergenic | Olfr811 | ENSMUSG00000063173 |
| chr6 | 134250297 | 134250319 | + | 4 | CACCCAGATCAAAAGAAAAT | GGG | | 1493 | Intronic | Etv6 | ENSMUSG00000030199 |
| chr3 | 120119547 | 120119569 | − | 4 | CCCACTAATTAAACAAAAAT | AGG | NA | | Intergenic | NA | NA |
| chrX | 113387656 | 113387678 | − | 4 | CTCTCTCACCAAAATAAAAT | GGG | | 55431 | Intronic | Gm24831 | ENSMUSG00000080428 |
| chr6 | 51272825 | 51272847 | + | 3 | CACACTGAGCAAAAGAAAAT | AGG | | 2915 | Intergenic | Mir148a | ENSMUSG00000065505 |
| chr18 | 65971111 | 65971133 | − | 4 | CACCATCATCACCAAAAAAT | AGG | | 933 | Intergenic | Cplx4 | ENSMUSG00000024519 |
| chr5 | 76442127 | 76442149 | + | 4 | AGCACTCATAAAAACAAAAT | TGG | | 8552 | Intergenic | Gm42665 | ENSMUSG00000106982 |
| chr8 | 54271199 | 54271221 | + | 4 | ACCACTCATGAAAACAAAAT | TGG | | 84745 | Intergenic | Vegfc | ENSMUSG00000031520 |
| chr10 | 62635557 | 62635579 | − | 4 | CACATGCATAAAATAAAAAT | AGG | | 1431 | Intronic | Ddx50 | ENSMUSG00000020076 |
| chr14 | 87082295 | 87082317 | + | 3 | CACACACATTAAAATAAAAT | AGG | | 8661 | Intronic | Diap3 | ENSMUSG00000022021 |
| chr19 | 24622389 | 24622411 | − | 3 | CACACACATTAAAATAAAAT | AGG | | 51597 | Intergenic | Tmem252 | ENSMUSG00000048572 |
| chr7 | 73075279 | 73075301 | − | 4 | CACACAAATCCACAAAAAAT | GGG | | 14122 | Intergenic | RP24-136L4.3 | ENSMUSG00000108329 |
| chr2 | 61969874 | 61969896 | + | 4 | CACACTACTGAATAAAAAAT | GGG | | 458 | Intergenic | Gm13555 | ENSMUSG00000083018 |
| chr14 | 96244853 | 96244875 | + | 4 | AAAACTAATCAAAAAGAAAT | TGG | | 4459 | Intronic | Klhl1 | ENSMUSG00000022076 |
| chr17 | 77080627 | 77080649 | − | 4 | AAAACTGATCAAAAAGAAAT | AGG | NA | | Intergenic | NA | NA |
| chrX | 42143126 | 42143148 | + | 4 | CAGACTTCTCAAAACAAAAT | TGG | | 4840 | Intergenic | Gm37564 | ENSMUSG00000104042 |
| chr1 | 110268779 | 110268801 | − | 4 | CACAAACATCATTAAAAAAT | TGG | NA | | Intergenic | NA | NA |
| chr7 | 64289198 | 64289220 | + | 4 | CACAATAATTAAAGAAAAAT | CGG | | 799 | Intronic | Mtmr10 | ENSMUSG00000030522 |
| chrX | 22201803 | 22201825 | − | 4 | AACAATCATAAAAATAAAAT | AGG | | 3345 | Intergenic | Gm14563 | ENSMUSG00000082175 |
| chr8 | 39755438 | 39755460 | + | 4 | CACAAGCAACAAAAGAAAAT | AGG | NA | | Intergenic | NA | NA |
| chr18 | 88965295 | 88965317 | + | 4 | CATACACATAAAAATAAAAT | AGG | | 6473 | Intergenic | Rttn | ENSMUSG00000023066 |
| chr1 | 32967777 | 32967799 | + | 3 | TACACGCATCAAAAAATAAT | TGG | | 0 | Exonic | Gm37151 | ENSMUSG00000102926 |
| chr18 | 58506909 | 58506931 | + | 4 | AACAGTCCTCAAAAATAAAT | GGG | | 49326 | Intergenic | Slc27a6 | ENSMUSG00000024600 |
| chr5 | 102397266 | 102397288 | + | 4 | CACTGTGATCAAAAACAAAT | TGG | | 18915 | Intergenic | Gm42932 | ENSMUSG00000106295 |
| chr8 | 103567847 | 103567869 | − | 4 | CACATTTAACAAAACAAAAT | GGG | NA | | Intergenic | NA | NA |
| chr8 | 33005176 | 33005198 | + | 4 | CATATTCTTCAAAAAGAAAT | AGG | | 55150 | Intergenic | Gm3985 | ENSMUSG00000079070 |
| chr10 | 92597307 | 92597329 | − | 4 | CACAATCCACAAAACAAAAT | TGG | | 10000 | Intergenic | Gm22582 | ENSMUSG00000076238 |
| chr6 | 18382266 | 18382288 | − | 4 | CACAGTAATCAGAGAAAAAT | AGG | | 458 | Intronic | Cttnbp2 | ENSMUSG00000000416 |
| chr14 | 73986018 | 73986040 | − | 4 | TACACACACCAAAAAGAAAT | AGG | | 30467 | Intergenic | Cbx3-ps6 | ENSMUSG00000057886 |
| chr14 | 61743550 | 61743572 | + | 4 | CAGATTCACCAAAAACAAAT | AGG | | 57894 | Intergenic | Gm37820 | ENSMUSG00000104187 |
| chr9 | 47797088 | 47797110 | − | 4 | GACATTCATCATAAGAAAAT | GGG | | 297 | Intronic | Cadm1 | ENSMUSG00000032076 |
| chr10 | 15886115 | 15886137 | − | 4 | CACAATCTTTAAAAGAAAAT | AGG | NA | | Intergenic | NA | NA |
| chr4 | 92324975 | 92324997 | + | 4 | GACCCCCATCAAAAAAGAAT | AGG | | 48054 | Intergenic | Gm12638 | ENSMUSG00000083296 |
| chr14 | 80416664 | 80416686 | + | 4 | TACACACATCAGAACAAAAT | AGG | | 74179 | Intergenic | Gm9578 | ENSMUSG00000098125 |
| chr5 | 81444339 | 81444361 | − | 4 | CTCATTCATGAAAAAGAAAT | TGG | | 20834 | Intronic | Adgrl3 | ENSMUSG00000037605 |
| chr9 | 63117106 | 63117128 | − | 4 | AACACTGATCACAAGAAAAT | GGG | | 16155 | Intergenic | Gm25064 | ENSMUSG00000094201 |
| chr3 | 19776589 | 19776611 | + | 4 | CACAACCAGCAAAAATAAAT | AGG | | 7334 | Intergenic | Gm7442 | ENSMUSG00000106182 |
| chr3 | 158473852 | 158473874 | + | 4 | CTCACTAATTAAAACAAAAT | AGG | | 15117 | Intronic | Gm43487 | ENSMUSG00000105683 |
| chr3 | 106374685 | 106374707 | + | 4 | CAAAGTTATCAAAAAAGAAT | TGG | | 378 | Intergenic | Gm43212 | ENSMUSG00000105436 |
| chr1 | 186065171 | 186065193 | − | 4 | AACACTCTTAAAAAATAAAT | AGG | | 22538 | Intergenic | Lyplal1 | ENSMUSG00000039246 |
| chrX | 114886348 | 114886370 | + | 4 | CAAAGTCATCAACATAAAAT | TGG | | 18642 | Intergenic | Ube2dnl1 | ENSMUSG00000050435 |
| chr4 | 127593345 | 127593367 | + | 3 | CACACACACCAAAAAATAAT | AGG | | 22843 | Intergenic | A630031M04Ri | ENSMUSG00000087470 |
| chr15 | 64037388 | 64037410 | + | 4 | CACATTCCACAAAAATAAAT | GGG | | 11336 | Intronic | Gm25628 | ENSMUSG00000065936 |
| chr18 | 72323672 | 72323694 | + | 4 | CCTACTCATCATAAACAAAT | AGG | | 26662 | Intronic | Dcc | ENSMUSG00000060534 |
| chrX | 147457301 | 147457323 | − | 4 | CACAATAATAAAAAATAAAT | TGG | | 3594 | Intergenic | Vmn1r239-ps | ENSMUSG00000067262 |
| chrX | 109677819 | 109677841 | + | 4 | CTCTCTCATCAGAAACAAAT | GGG | NA | | Intergenic | NA | NA |
| chr1 | 185420854 | 185420876 | + | 4 | CACACCCCACAAAAATAAAT | GGG | | 567 | Intronic | Eprs | ENSMUSG00000026615 |
| chr18 | 20930451 | 20930473 | + | 4 | CACACCCCACAAAAATAAAT | AGG | | 14152 | Intergenic | Rnf125 | ENSMUSG00000033107 |
| chr6 | 30408069 | 30408091 | + | 4 | CACTCTCTTAAAAAAGAAAT | AGG | | 5919 | Intronic | Klhdc10 | ENSMUSG00000029775 |
| chr5 | 132604077 | 132604099 | − | 4 | CACACACCTTAAAAAGAAAT | AGG | | 53444 | Intergenic | Gm42626 | ENSMUSG00000105809 |
| chr4 | 36774309 | 36774331 | − | 4 | TACAGTCATTAAAAAACAAT | TGG | | 18155 | Intronic | Gm23314 | ENSMUSG00000087843 |
| chr15 | 54035583 | 54035605 | + | 3 | CACACTCCTCAAAGTAAAAT | TGG | | 0 | Exonic | Gm26933 | ENSMUSG00000097938 |

| | | | | | | | | | | | |
|---|---|---|---|---|---|---|---|---|---|---|---|
| chr2 | 158276185 | 158276207 | + | 4 | AACACTGATAAAAAAAGAAT | GGG | | 196 | Intronic | Bpi | ENSMUSG00000052922 |
| chr5 | 53858375 | 53858397 | − | 4 | CATACTCTTTAAAAAAGAAT | AGG | | 1453 | Intronic | Tbc1d19 | ENSMUSG00000039178 |
| chr1 | 161545010 | 161545032 | − | 4 | CAAAGTTATCAAAAAAAGAT | AGG | | 7517 | Intergenic | Gm25488 | ENSMUSG00000089353 |
| chr10 | 126373434 | 126373456 | − | 4 | TACATTCATCAAATATAAAT | CGG | NA | | Intergenic | NA | NA |
| chr9 | 6582992 | 6583014 | − | 4 | CACACTGTTCATAAAGAAAT | AGG | NA | | Intergenic | NA | NA |
| chr17 | 72484749 | 72484771 | − | 4 | CTGACTCATCAATAAATAAT | TGG | | 14518 | Intronic | Gm24736 | ENSMUSG00000089487 |
| chr13 | 89988220 | 89988242 | + | 4 | CACAGCCATCACAAAAGAAT | AGG | | 2831 | Intronic | Xrcc4 | ENSMUSG00000021615 |
| chrX | 48088706 | 48088728 | + | 4 | CACAGCCATCAAACAGAAAT | GGG | | 19997 | Intergenic | Xpnpep2 | ENSMUSG00000037005 |
| chr5 | 120736718 | 120736740 | − | 4 | CACATTTGTCAAAAAAACAT | AGG | | 534 | Intronic | Oas2 | ENSMUSG00000032690 |
| chr18 | 5454256 | 5454278 | − | 4 | CCCACACATCAACAAAGAAT | AGG | | 37225 | Intergenic | Gm10125 | ENSMUSG00000063087 |
| chr18 | 9667604 | 9667626 | − | 4 | GACACTAATGAAAAAAATAT | TGG | | 40022 | Intergenic | Colec12 | ENSMUSG00000036103 |
| chr12 | 4081925 | 4081947 | − | 4 | CTTACACATCAAAAAAAATT | TGG | | 627 | Intergenic | Dnajc27 | ENSMUSG00000020657 |
| chr11 | 13589042 | 13589064 | − | 4 | AACACTGATCAAAATCAAAT | TGG | NA | | Intergenic | NA | NA |
| chr17 | 55644577 | 55644599 | + | 4 | AACAATCATCAAAGAAGAAT | TGG | | 7426 | Intergenic | Pot1b | ENSMUSG00000024174 |
| chr1 | 73602311 | 73602333 | + | 4 | CACAGACATCAAAACCAAAT | GGG | | 15755 | Intergenic | 6030407O03Ril | ENSMUSG00000100301 |
| chr15 | 50123984 | 50124006 | + | 4 | CACACATATGAAAAAAACAT | TGG | NA | | Intergenic | NA | NA |
| chr4 | 111230136 | 111230158 | + | 4 | CACCCTGATCAAAAGCAAAT | TGG | | 28162 | Intronic | Agbl4 | ENSMUSG00000061298 |
| chrX | 147134789 | 147134811 | + | 4 | CACAATAATCAAAAGCAAAT | TGG | | 23399 | Intronic | Mir448 | ENSMUSG00000065450 |
| chr7 | 142060650 | 142060672 | + | 4 | CACTCTCCTCAAAAGTAAAT | TGG | | 313 | Intronic | Mob2 | ENSMUSG00000025147 |
| chr2 | 26989725 | 26989747 | + | 4 | CACCCCCATCAGAAAAACAT | GGG | | 306 | Intronic | Adamts13 | ENSMUSG00000014852 |
| chr4 | 108120313 | 108120335 | − | 4 | CAGACTCTTCAAATAACAAT | CGG | | 1781 | Intronic | Scp2 | ENSMUSG00000028603 |
| chr15 | 89551327 | 89551349 | − | 3 | AACATTCATCAAAAAAAAAG | AGG | | 1445 | Intronic | Shank3 | ENSMUSG00000022623 |
| chr1 | 91588929 | 91588951 | + | 4 | CAGACACATTAAAAAAAAGT | GGG | | 22455 | Intergenic | Gm28380 | ENSMUSG00000101705 |
| chr5 | 146928876 | 146928898 | − | 3 | CACCATCATCAAAAAAAAAA | TGG | | 19759 | Intergenic | Gtf3a | ENSMUSG00000016503 |
| chr8 | 54801849 | 54801871 | − | 4 | CAAACTGATCAAAACAGAAT | GGG | | 26427 | Intronic | Wdr17 | ENSMUSG00000039375 |
| chr15 | 21090457 | 21090479 | − | 4 | GTCTCTCATCAAAAAAAAAC | AGG | | 20973 | Intergenic | Cdh12 | ENSMUSG00000040452 |
| chrX | 136984738 | 136984760 | − | 4 | CACACATACCAAAAAAAATT | TGG | | 1117 | Intronic | Slc25a53 | ENSMUSG00000044348 |
| chr1 | 60467108 | 60467130 | − | 4 | CACACAAACCAAAAAAAAGT | AGG | | 2493 | Intronic | Abi2 | ENSMUSG00000026782 |
| chrX | 150869169 | 150869191 | + | 4 | AAAACTCATCAGAAAAAAGT | AGG | | 12116 | Intergenic | Itih5l-ps | ENSMUSG00000087149 |
| chr2 | 27409706 | 27409728 | + | 4 | CACACCAATTAAAAAAAACT | GGG | | 7755 | Intronic | Gm24049 | ENSMUSG00000076364 |
| chr12 | 16522944 | 16522966 | − | 4 | CAAGCCCATCAAAAAAAAAA | TGG | | 12703 | Intergenic | Lpin1 | ENSMUSG00000020593 |
| chr2 | 85979679 | 85979701 | + | 4 | CACTGTCATCAAAAATTAAT | GGG | | 3460 | Intergenic | Olfr1029 | ENSMUSG00000059873 |
| chr16 | 57748992 | 57749014 | + | 4 | AGCACTCTTCAAAAAAAAAA | AGG | | 5626 | Intronic | Col8a1 | ENSMUSG00000068196 |
| chrY | 59626923 | 59626945 | + | 4 | CAAAGTTATCAAAAAAAAAG | AGG | | 1018 | Intronic | Gm20871 | ENSMUSG00000101005 |
| chrY | 38794956 | 38794978 | + | 4 | CAAAGTTATCAAAAAAAAAG | AGG | | 1018 | Intronic | Gm20901 | ENSMUSG00000096275 |
| chrY | 19118837 | 19118859 | − | 4 | CAAAGTTATCAAAAAAAAAG | AGG | | 1018 | Intronic | Gm35134 | ENSMUSG00000103371 |
| chrY | 14406541 | 14406563 | − | 4 | CAAAGTTATCAAAAAAAAAG | AGG | | 1018 | Intronic | Gm30737 | ENSMUSG00000104191 |
| chrY | 42936138 | 42936160 | − | 4 | CAAAGTTATCAAAAAAAAAG | AGG | | 1018 | Intronic | Gm29165 | ENSMUSG00000099833 |
| chrY | 40311046 | 40311068 | + | 4 | CAAAGTTATCAAAAAAAAAG | AGG | | 1019 | Intronic | Gm21865 | ENSMUSG00000093895 |
| chrY | 43957992 | 43958014 | − | 4 | CAAAGTTATCAAAAAAAAAG | AGG | | 1019 | Intronic | Gm21241 | ENSMUSG00000096332 |
| chrY | 31377802 | 31377824 | − | 4 | CAAAGTTATCAAAAAAAAAG | AGG | | 1019 | Intronic | Gm21737 | ENSMUSG00000094623 |
| chrY | 33250843 | 33250865 | − | 4 | CAAAGTTATCAAAAAAAAAG | AGG | | 1019 | Intronic | Gm21756 | ENSMUSG00000095049 |
| chrY | 4789063 | 4789085 | + | 4 | CAAAGTTATCAAAAAAAAAG | AGG | | 1020 | Intronic | Gm28356 | ENSMUSG00000099684 |
| chrY | 29356220 | 29356242 | − | 4 | CAAAGTTATCAAAAAAAAAG | AGG | | 1020 | Intronic | Gm21853 | ENSMUSG00000096538 |
| chrY | 40925255 | 40925277 | + | 4 | CAAAGTTATCAAAAAAAAAC | AGG | | 1024 | Intronic | Gm20832 | ENSMUSG00000099645 |
| chrY | 18506526 | 18506548 | − | 4 | CAAAGTTATCAAAAAAAAAC | AGG | | 1024 | Intronic | Gm30638 | ENSMUSG00000102264 |
| chrY | 13793803 | 13793825 | − | 4 | CAAAGTTATCAAAAAAAAAC | AGG | | 1024 | Intronic | Gm36728 | ENSMUSG00000104404 |
| chrY | 11698233 | 11698255 | − | 4 | CAAAGTTATCAAAAAAAAAG | AGG | | 1026 | Intronic | Gm28951 | ENSMUSG00000099572 |
| chrY | 16383628 | 16383650 | − | 4 | CAAAGTTATCAAAAAAAAAG | AGG | | 1026 | Intronic | Gm32033 | ENSMUSG00000102208 |
| chrY | 68826897 | 68826919 | + | 4 | CAAAGTTATCAAAAAAAAAG | AGG | | 1035 | Intronic | Gm20817 | ENSMUSG00000100032 |
| chrY | 57193019 | 57193041 | − | 4 | CAAAGTTATCAAAAAAAAAG | AGG | | 1036 | Intronic | Sly | ENSMUSG00000101155 |
| chrY | 76767411 | 76767433 | − | 4 | CAAAGTTATCAAAAAAAAAG | AGG | | 1036 | Intronic | Gm21173 | ENSMUSG00000100708 |
| chrY | 61652557 | 61652579 | − | 4 | CAAAGTTATCAAAAAAAAAG | AGG | | 1038 | Intronic | Gm21497 | ENSMUSG00000100726 |
| chrY | 48862308 | 48862330 | − | 4 | CAAAGTTATCAAAAAAAAAG | AGG | | 1039 | Intronic | Gm28553 | ENSMUSG00000100972 |
| chr6 | 79828059 | 79828081 | + | 4 | CAAAGTTATCAAAAAAAAAA | TGG | | 9695 | Intergenic | Gm20594 | ENSMUSG00000096887 |
| chr3 | 67641770 | 67641792 | − | 4 | CAAAGTTATCAAAAAAAAAG | GGG | | 21042 | Intergenic | Gm412 | ENSMUSG00000103606 |
| chr1 | 113233971 | 113233993 | + | 4 | CAAAGTTATCAAAAAAAAAA | TGG | | 55347 | Intergenic | Gm28189 | ENSMUSG00000101453 |
| chrX | 156116296 | 156116318 | + | 3 | CACATTCATGAAAAAAAAAC | TGG | | 54497 | Intronic | Gm15155 | ENSMUSG00000055109 |
| chr1 | 61640835 | 61640857 | + | 4 | CCCACACTTCAAAAAAAAAA | AGG | | 1473 | Intronic | Pard3b | ENSMUSG00000052062 |
| chr6 | 149499811 | 149499833 | + | 4 | CACAACAATCAAAAAAAAAA | TGG | | 2040 | Intronic | Bicd1 | ENSMUSG00000003452 |
| chr4 | 144353936 | 144353958 | − | 4 | CACAACAATCAAAAAAAAAA | TGG | | 3984 | Intergenic | Gm13119 | ENSMUSG00000070619 |
| chr2 | 87404403 | 87404425 | + | 4 | CACAACAATCAAAAAAAAAA | TGG | | 9759 | Intergenic | Gm1826 | ENSMUSG00000082028 |
| chr8 | 66602928 | 66602950 | − | 4 | CACAACAATCAAAAAAAAAA | TGG | | 13920 | Intergenic | Gm16330 | ENSMUSG00000085082 |
| chr18 | 17512983 | 17513005 | + | 4 | CACAACAATCAAAAAAAAAA | TGG | NA | | Intergenic | NA | NA |
| chr15 | 98663408 | 98663430 | − | 4 | AACACTTTTCAAAAAAAAAG | GGG | | 0 | Exonic | Rnd1 | ENSMUSG00000054855 |
| chr1 | 103227170 | 103227192 | − | 4 | TAAACTCATTAAAAAAAAAA | AGG | | 9434 | Intronic | Gm23965 | ENSMUSG00000088594 |
| chr16 | 93905611 | 93905633 | − | 3 | CACACACATTAAAAAAAAAA | AGG | | 272 | Intronic | Chaf1b | ENSMUSG00000022945 |
| chr7 | 41077274 | 41077296 | + | 3 | CACACACATTAAAAAAAAAA | AGG | | 329 | Intronic | RP23-472J11.1 | ENSMUSG00000108619 |
| chr16 | 90141907 | 90141929 | + | 3 | CACACGCATAAAAAAAAAAA | AGG | | 78813 | Intergenic | Sod1 | ENSMUSG00000022982 |
| chr17 | 41730737 | 41730759 | − | 3 | CACACACATGAAAAAAAAAA | AGG | NA | | Intergenic | NA | NA |
| chrX | 9915487 | 9915509 | − | 4 | GACCCTCATGAAAAAAAAAA | AGG | | 0 | Exonic | Sytl5 | ENSMUSG00000054453 |
| chr4 | 31771808 | 31771830 | + | 4 | CTCTCTCATAAAAAAAAAAG | AGG | NA | | Intergenic | NA | NA |
| chrX | 145457842 | 145457864 | + | 3 | CACACTTATTAAAAAAAAAC | TGG | | 1884 | Intronic | Amot | ENSMUSG00000041688 |
| chr2 | 172733247 | 172733269 | + | 4 | CAAACGCAACAAAAAAAAAA | AGG | NA | | Intergenic | NA | NA |
| chr16 | 16779619 | 16779641 | + | 4 | GCCACTCATCTAAAAAAAAC | TGG | | 980 | Intronic | Spag6 | ENSMUSG00000022783 |
| chrX | 14303246 | 14303268 | − | 4 | TACATTCATCAAAGAAAACT | AGG | | 12133 | Intergenic | Gm25202 | ENSMUSG00000064772 |
| chr13 | 47316461 | 47316483 | − | 4 | AACAATCATCAACAAAATT | AGG | | 68470 | Intergenic | Rnf144b | ENSMUSG00000038068 |
| chr10 | 90701074 | 90701096 | + | 4 | CACAGTAGTCAAAAAAAAAA | AGG | | 17377 | Intronic | Anks1b | ENSMUSG00000058589 |
| chr14 | 13950656 | 13950678 | + | 4 | AACACCCATTAAAAAAAAAA | AGG | | 291 | Intronic | Thoc7 | ENSMUSG00000053453 |
| chr13 | 102634963 | 102634985 | + | 4 | AACACACATTAAAAAAAAAA | AGG | | 58573 | Intergenic | Cd180 | ENSMUSG00000021624 |
| chr12 | 61784194 | 61784216 | − | 4 | CAAAATCATTAAAAAAAAAG | TGG | | 55192 | Intronic | Lrfn5 | ENSMUSG00000035653 |
| chr7 | 79276899 | 79276921 | − | 4 | CACAATCCTCAAAAGAAGAT | GGG | | 846 | Intronic | RP23-256J14.1 | ENSMUSG00000108521 |
| chr10 | 31454983 | 31455005 | + | 4 | CACTGTCATTAAAAAAAAAA | AGG | | 9062 | Intergenic | Tpd52l1 | ENSMUSG00000000296 |
| chr19 | 53553968 | 53553990 | + | 4 | CATACACATAAAAAAAAAAA | AGG | | 11537 | Intergenic | Dusp5 | ENSMUSG00000034765 |
| chr13 | 112781048 | 112781070 | + | 4 | AACAATCATCCAAAAAAAAG | AGG | | 19824 | Intergenic | Ppap2a | ENSMUSG00000021759 |

| | | | | | | | | | | | |
|---|---|---|---|---|---|---|---|---|---|---|---|
| chr7 | 131441362 | 131441384 | − | 4 | CTCACTTATTAAAAAAAAAG | AGG | | 23 | Intronic | Acadsb | ENSMUSG00000030861 |
| chr17 | 26233111 | 26233133 | + | 4 | CGCACTGATAAAAAAAAAAA | GGG | | 7088 | Intronic | Fam234a | ENSMUSG00000024187 |
| chr3 | 126479015 | 126479037 | + | 4 | CTCACTTATAAAAAAAAAAG | TGG | | 31065 | Intergenic | Gm20568 | ENSMUSG00000105233 |
| chr3 | 140133819 | 140133841 | + | 4 | GACACTTATCAAACAAAATT | GGG | | 15784 | Intergenic | Gm4863 | ENSMUSG00000105180 |
| chr4 | 102357257 | 102357279 | − | 4 | CAAACTGATAAAAAAAAAAA | TGG | | 64239 | Intronic | Pde4b | ENSMUSG00000028525 |
| chr4 | 119032956 | 119032978 | − | 4 | CACATACATGAAAAAAAAAA | AGG | | 21812 | Intergenic | Gm12866 | ENSMUSG00000066060 |
| chr7 | 77680261 | 77680283 | − | 4 | CACAACCATGAAAAAAAAAA | AGG | NA | | Intergenic | NA | NA |
| chr15 | 37246253 | 37246275 | − | 4 | AACACTCTTAAAAAAAAAAA | AGG | | 3091 | Intronic | Gm16137 | ENSMUSG00000089949 |
| chr17 | 70786706 | 70786728 | + | 3 | CACACCCATCAAAACAAACT | GGG | | 60 | Intronic | Dlgap1 | ENSMUSG00000003279 |
| chr5 | 62065005 | 62065027 | + | 4 | CAAACTCCTAAAAAAAAAAA | AGG | | 60170 | Intergenic | Gm42430 | ENSMUSG00000106530 |
| chr8 | 44205307 | 44205329 | − | 4 | CACAATGATAAAAAAAAAAA | TGG | | 22389 | Intergenic | Gm37972 | ENSMUSG00000102378 |
| chrX | 20825562 | 20825584 | − | 4 | CACACAGATGAAAAAAAAAA | TGG | | 1748 | Intronic | Gm24824 | ENSMUSG00000092838 |
| chrX | 159436356 | 159436378 | − | 4 | CACACATATGAAAAAAAAAC | AGG | | 7545 | Intronic | Map7d2 | ENSMUSG00000041020 |
| chr11 | 72422986 | 72423008 | + | 4 | CACACAAATTAAAAAAAAAA | AGG | | 11273 | Intergenic | Smtnl2 | ENSMUSG00000045667 |
| chr13 | 53233058 | 53233080 | − | 4 | CACACAAATGAAAAAAAAAA | GGG | | 42411 | Intronic | Ror2 | ENSMUSG00000021464 |
| chr16 | 82872720 | 82872742 | + | 4 | CACACTGGACAAAAAAAAAA | AGG | | 42839 | Intergenic | Gm21833 | ENSMUSG00000094030 |
| chr16 | 38543501 | 38543523 | − | 4 | CACACACTTTAAAAAAAAAA | AGG | | 542 | Intronic | Poglut1 | ENSMUSG00000034064 |
| chr3 | 74333603 | 74333625 | − | 4 | CACCATCATCATAAAAAAAG | GGG | | 9611 | Intergenic | Gm37050 | ENSMUSG00000104227 |
| chr8 | 15742126 | 15742148 | + | 4 | AATACTCATCAACAAAAAAC | TGG | NA | | Intergenic | NA | NA |
| chr13 | 99664774 | 99664796 | + | 4 | CAAACACATCAGAAAAAAAA | AGG | | 21971 | Intergenic | 1700012J22Rik | ENSMUSG00000103545 |
| chr2 | 57612319 | 57612341 | + | 4 | GACACTAATCACAAAAAAAC | AGG | | 16648 | Intergenic | Gm13532 | ENSMUSG00000081642 |
| chr6 | 123962180 | 123962202 | + | 4 | AACACTAATCAAAAGAAAGT | TGG | | 1431 | Intronic | A330044P14Rik | ENSMUSG00000093385 |
| chr6 | 123831969 | 123831991 | + | 4 | AACACTAATCAAAAGAAAGT | TGG | | 3399 | Intronic | Vmn2r25 | ENSMUSG00000094672 |
| chr6 | 124047194 | 124047216 | − | 4 | AACACTAATCAAAAGAAAGT | TGG | | 3413 | Intronic | Vmn2r26 | ENSMUSG00000096630 |
| chr1 | 134267058 | 134267080 | + | 4 | AACACTAATCAAAAGAAAGT | TGG | | 13805 | Intergenic | Gm24517 | ENSMUSG00000096676 |
| chr9 | 102418601 | 102418623 | − | 4 | CAGACTAATCAAAATAAACT | AGG | | 45381 | Intergenic | Gm22894 | ENSMUSG00000088138 |
| chr1 | 94176464 | 94176486 | − | 3 | CATACTCATCAAAGAAAAAC | TGG | | 27931 | Intergenic | Gm7891 | ENSMUSG00000100990 |
| chr13 | 66427358 | 66427380 | + | 4 | CGCACTCCTCAAAAGAAAGT | GGG | | 2340 | Intronic | 2410141K09Rik | ENSMUSG00000074832 |
| chr13 | 66223498 | 66223520 | + | 4 | CGCACTCCTCAAAAGAAAGT | GGG | | 3799 | Intergenic | Gm10772 | ENSMUSG00000091347 |
| chr13 | 65543206 | 65543228 | + | 4 | CGCACTCCTCAAAAGAAAGT | GGG | | 15938 | Intergenic | Cbx3-ps2 | ENSMUSG00000095692 |
| chr7 | 45925775 | 45925797 | − | 4 | CAGACTCTTCAGAAAAAAAA | GGG | | 708 | Intronic | Ccdc114 | ENSMUSG00000040189 |
| chr18 | 70745395 | 70745417 | − | 4 | CACCCTCCTCATAAAAAAAA | AGG | NA | | Intergenic | NA | NA |
| chr5 | 74082331 | 74082353 | − | 4 | ATCACTCATCAAATAAAAAG | CGG | | 9784 | Intergenic | Gm43416 | ENSMUSG00000107255 |
| chr8 | 105397044 | 105397066 | − | 4 | CACACCAATCATAAAAAAAG | TGG | | 564 | Intronic | Kctd19 | ENSMUSG00000051648 |
| chr13 | 54699749 | 54699771 | − | 4 | CACACGAATCAAAAGAAACT | GGG | | 1690 | Intergenic | Cdhr2 | ENSMUSG00000034918 |
| chr6 | 86256835 | 86256857 | − | 4 | TACACTCCTCAAAAAACTAT | AGG | | 4698 | Intronic | RP23-304D1.6 | ENSMUSG00000107745 |
| chr4 | 122290683 | 122290705 | − | 4 | AACACTCTTCAACAAAAAAG | AGG | | 54766 | Intergenic | Gm12893 | ENSMUSG00000083867 |
| chr4 | 121897697 | 121897719 | − | 4 | AACACTCTTCAACAAAAAAG | AGG | NA | | Intergenic | NA | NA |
| chr4 | 109343906 | 109343928 | − | 3 | CACACTTATCAAATAAAAAC | AGG | | 550 | Intronic | Eps15 | ENSMUSG00000028552 |
| chrX | 114446060 | 114446082 | − | 4 | AACACTGATCAAAAATAACT | TGG | | 28251 | Intergenic | Klhl4 | ENSMUSG00000025597 |
| chr8 | 127102552 | 127102574 | + | 4 | AACACTGATCAAAAACAAGT | TGG | | 29266 | Intronic | Pard3 | ENSMUSG00000025812 |
| chr3 | 64987022 | 64987044 | + | 4 | AACACTGATCAAAAATAACT | TGG | | 37489 | Intronic | Gm38048 | ENSMUSG00000102437 |
| chr3 | 112134394 | 112134416 | + | 4 | AACACTAATCAAAAACAACT | TGG | | 52393 | Intergenic | Gm6602 | ENSMUSG00000104543 |
| chr9 | 10844861 | 10844883 | + | 4 | CACACTGGTCAAAATAAATT | TGG | | 59477 | Intronic | Cntn5 | ENSMUSG00000039488 |
| chr11 | 29031617 | 29031639 | + | 4 | CACTTTCATCAAAGAAAAAA | TGG | | 74009 | Intergenic | Rpsa-ps5 | ENSMUSG00000081695 |
| chr1 | 30465491 | 30465513 | + | 4 | AACACTAATCAAAGAAAAAG | AGG | NA | | Intergenic | NA | NA |
| chr9 | 15744290 | 15744312 | + | 4 | CACCCTGATCAAAAACAACT | TGG | | 1906 | Intergenic | Slc36a4 | ENSMUSG00000043885 |
| chr13 | 75556457 | 75556479 | + | 4 | CACAATAATCAAAAACAACT | TGG | | 88385 | Intergenic | Gm4149 | ENSMUSG00000074800 |
| chr10 | 33220319 | 33220341 | − | 4 | CACTCTCTTCAAAAATAACT | TGG | | 13514 | Intronic | Trdn | ENSMUSG00000019787 |
| chr10 | 33038926 | 33038948 | + | 4 | CACTCTCCTCAAAAATAACT | TGG | | 44535 | Intergenic | Trdn | ENSMUSG00000019787 |
| chr4 | 80707874 | 80707896 | − | 4 | CACTCTCCTCAAAAATAACT | TGG | NA | | Intergenic | NA | NA |
| chr4 | 85254397 | 85254419 | + | 4 | CACAATAATCAAAGAAAAAG | AGG | | 8635 | Intronic | Gm12413 | ENSMUSG00000081862 |
| chr9 | 44233305 | 44233327 | − | 4 | CACACAGATCAAAGAAAAAG | AGG | | 34 | Intronic | Cbl | ENSMUSG00000034342 |
| chr3 | 13663517 | 13663539 | − | 4 | CACACAAATCAAAGAAAAAG | AGG | | 5970 | Intergenic | Gm37164 | ENSMUSG00000104514 |
| chr2 | 87293013 | 87293035 | + | 4 | CATATTCATCAAAAGAAAAA | AGG | | 7741 | Intergenic | Olfr1117-ps1 | ENSMUSG00000083706 |
| chr5 | 48692365 | 48692387 | + | 4 | CAAAGTCATCAAAACAAAAA | AGG | | 19703 | Intronic | Gm43829 | ENSMUSG00000105530 |
| chr7 | 18361784 | 18361806 | + | 4 | CAAAGTCATCAAAAAAGACT | AGG | | 6775 | Intergenic | Psg18 | ENSMUSG00000003505 |
| chr6 | 106363460 | 106363482 | − | 4 | CAAAGTCATCAAAAAAGACT | AGG | | 9680 | Intronic | Cntn4 | ENSMUSG00000064293 |
| chr17 | 68547417 | 68547439 | − | 4 | CAAAGTCATCAAAAAAGAGT | AGG | | 12310 | Intronic | L3mbtl4 | ENSMUSG00000041565 |
| chr10 | 33694022 | 33694044 | − | 4 | CAAAGTCATCAAAAAAGACT | AGG | | 31590 | Intergenic | Gm15939 | ENSMUSG00000090087 |
| chr13 | 88528216 | 88528238 | − | 4 | CAAAGTCATCAAAAAAGACT | AGG | | 49160 | Intergenic | Gm8526 | ENSMUSG00000057685 |
| chr16 | 69704203 | 69704225 | − | 4 | CAAAGTCATCAAAAAAGACT | AGG | | 91910 | Intergenic | Gm43265 | ENSMUSG00000107073 |
| chr16 | 27693333 | 27693355 | + | 4 | CAAAGTCATCAAAAAAGACT | AGG | NA | | Intergenic | NA | NA |
| chr8 | 54318756 | 54318778 | + | 4 | CAAAGTCATCAAAAAAGACT | AGG | NA | | Intergenic | NA | NA |
| chr16 | 51175994 | 51176016 | + | 4 | CAAAGTCATCAAAAAAGACT | AGG | NA | | Intergenic | NA | NA |
| chr15 | 33726465 | 33726487 | − | 4 | CAAACTTATCAAAAGAAAAG | GGG | | 38582 | Intergenic | Tspyl5 | ENSMUSG00000038984 |
| chr4 | 114151879 | 114151901 | − | 4 | CAAACTTATCAAAAAAGATT | AGG | | 11483 | Intergenic | Skint11 | ENSMUSG00000057977 |
| chr8 | 94490493 | 94490515 | − | 4 | CAGACTCTTCAAAAGAAAAC | AGG | | 71 | Intronic | Nlrc5 | ENSMUSG00000074151 |
| chr7 | 58305221 | 58305243 | − | 4 | CTCACACATCAAAAAGAAAA | TGG | NA | | Intergenic | NA | NA |
| chr2 | 98411426 | 98411448 | + | 3 | CAAACTCATCAAAAAAGAAA | AGG | NA | | Intergenic | NA | NA |
| chr5 | 18262360 | 18262382 | + | 4 | ATCACTCATCAAAAAAGAAC | AGG | | 2753 | Intergenic | Gnai1 | ENSMUSG00000057614 |
| chr7 | 87885576 | 87885598 | + | 4 | TGCACTCATCAAAAAAGAAC | TGG | | 75956 | Intronic | Grm5 | ENSMUSG00000049583 |
| chr5 | 46627962 | 46627984 | + | 4 | AAAACTCATCAAAAAAGAAA | AGG | NA | | Intergenic | NA | NA |
| chr14 | 16703542 | 16703564 | + | 4 | TAAACTCATCAAAAAATAAG | AGG | NA | | Intergenic | NA | NA |
| chr17 | 22382670 | 22382692 | + | 4 | CCCAGTCATCAAAAAAGAAA | AGG | | 5389 | Intergenic | Zfp758 | ENSMUSG00000044501 |
| chr13 | 8039218 | 8039240 | + | 4 | CAAAGTCATCAAAAAAGAAA | AGG | | 1112 | Intergenic | Gm9742 | ENSMUSG00000091053 |
| chr9 | 91219517 | 91219539 | + | 4 | CAAAGTCATCAAAAAAGAAA | AGG | | 3061 | Intergenic | Gm29602 | ENSMUSG00000100568 |
| chr10 | 100791681 | 100791703 | − | 4 | CAAAGTCATCAAAAAAGAAA | AGG | | 3458 | Intergenic | Gm38219 | ENSMUSG00000103550 |
| chr6 | 130468085 | 130468107 | + | 4 | CAAATTCATCAAAAAAGAAA | AGG | | 5463 | Intronic | RP23-451F7.8 | ENSMUSG00000107492 |
| chr6 | 68127317 | 68127339 | − | 4 | CAAAGTCATCAAAAAAGAAA | AGG | | 5490 | Intergenic | Igkv1-117 | ENSMUSG00000094335 |
| chr3 | 7544861 | 7544883 | + | 4 | CAAAGTCATCAAAAAAGAAA | AGG | | 5678 | Intronic | Zc2hc1a | ENSMUSG00000043542 |
| chr6 | 68277545 | 68277567 | − | 4 | CAAAGTCATCAAAAAAGAAA | AGG | | 6278 | Intergenic | Igkv1-110 | ENSMUSG00000093861 |
| chr2 | 36345315 | 36345337 | − | 4 | CAAAGTCATCAAAAAAGAAA | AGG | | 8235 | Intergenic | Olfr337-ps1 | ENSMUSG00000080936 |
| chrX | 17310692 | 17310714 | + | 4 | CAAAGTCATCAAAAAAGAAA | AGG | | 8528 | Intronic | Efhc2 | ENSMUSG00000025038 |
| chr19 | 39220318 | 39220340 | − | 4 | CAAAGTCATCAAAAAAGAAA | AGG | | 8914 | Intergenic | Cyp2c53-ps | ENSMUSG00000093610 |

| | | | | | | | | | | |
|---|---|---|---|---|---|---|---|---|---|---|
| chr3 | 139253209 | 139253231 | − | 4 | CAAAGTCATCAAAAAAGAAA | AGG | 10024 | Intronic | Stpg2 | ENSMUSG00000047940 |
| chrY | 1799198 | 1799220 | − | 4 | CAAAGTCATCAAAAAAGAAA | AGG | 10031 | Intergenic | Gm29167 | ENSMUSG00000101947 |
| chr6 | 132923297 | 132923319 | + | 4 | CAAAGTCATCAAAAAAGAAA | AGG | 10648 | Intergenic | Tas2r146-ps1 | ENSMUSG00000108261 |
| chrX | 62147330 | 62147352 | + | 4 | CAAAGTCATCAAAAAAGAAA | AGG | 11002 | Intergenic | Gm5390 | ENSMUSG00000083617 |
| chr8 | 7369840 | 7369862 | + | 4 | CAAAGTCATCAAAAAAGAAA | AGG | 11153 | Intergenic | Gm17215 | ENSMUSG00000092092 |
| chr8 | 129205129 | 129205151 | + | 4 | CAAAGTCATCAAAAAAGAAA | AGG | 11203 | Intergenic | 2610044O15Ril | ENSMUSG00000071302 |
| chr6 | 68400774 | 68400796 | + | 4 | CAAAGTCATCAAAAAAGAAA | AGG | 11703 | Intergenic | Gm5310 | ENSMUSG00000105248 |
| chrX | 13972621 | 13972643 | − | 4 | CAAAGTCATCAAAAAAGAAA | AGG | 12742 | Intergenic | Gm25552 | ENSMUSG00000088706 |
| chr7 | 11897306 | 11897328 | − | 4 | CAAAGTCATCAAAAAAGAAA | AGG | 13918 | Intergenic | Vmn1r-ps56 | ENSMUSG00000092285 |
| chr8 | 65143512 | 65143534 | − | 4 | CAAAGTCATCAAAAAAGAAA | AGG | 13949 | Intergenic | BC030870 | ENSMUSG00000074300 |
| chr17 | 38244910 | 38244932 | − | 4 | CAAAGTCATCAAAAAAGAAA | AGG | 16135 | Intergenic | Olfr138 | ENSMUSG00000057443 |
| chr7 | 10342485 | 10342507 | − | 4 | CAAAGTCATCAAAAAAGAAA | AGG | 16366 | Intergenic | Nlrp4d | ENSMUSG00000034122 |
| chr11 | 49388074 | 49388096 | + | 4 | CAAAGTCATCAAAAAAGAAA | AGG | 16524 | Intergenic | Gm12189 | ENSMUSG00000084278 |
| chrX | 4472675 | 4472697 | − | 4 | CAAAGTCATCAAAAAAGAAA | AGG | 16672 | Intergenic | Gm9427 | ENSMUSG00000080073 |
| chr19 | 33536418 | 33536440 | − | 4 | CAAAGTCATCAAAAAAGAAA | AGG | 18638 | Intronic | Lipo4 | ENSMUSG00000079344 |
| chrX | 90439336 | 90439358 | + | 4 | CAAAGTCATCAAAAAAGAAA | AGG | 19964 | Intergenic | Gm4746 | ENSMUSG00000082479 |
| chr6 | 66231929 | 66231951 | − | 4 | CAAAGTCATCAAAAAAGAAG | AGG | 22792 | Intergenic | RP23-478C7.1 | ENSMUSG00000107695 |
| chr1 | 150190960 | 150190982 | − | 4 | CAAAGTCATCAAAAAAGAAA | AGG | 26012 | Intergenic | Ptgs2os2 | ENSMUSG00000097754 |
| chr18 | 37589683 | 37589705 | + | 4 | CAAAGTCATCAAAAAAGAAA | AGG | 29201 | Intronic | 4930517L18Rik | ENSMUSG00000104225 |
| chr15 | 44315893 | 44315915 | + | 4 | CAAAGTCATCAAAAAAGAAA | AGG | 29735 | Intergenic | Gm24903 | ENSMUSG00000088756 |
| chr3 | 113089901 | 113089923 | + | 4 | CAAAGTCATCAAAAAAGAAA | AGG | 33276 | Intergenic | Gm38395 | ENSMUSG00000105665 |
| chrX | 137534494 | 137534516 | − | 4 | CAAAGTCATCAAAAAAGAAA | AGG | 36092 | Intergenic | Il1rapl2 | ENSMUSG00000059203 |
| chr8 | 63817555 | 63817577 | + | 4 | CAAAGTCATCAAAAAAGAAA | AGG | 38503 | Intergenic | Gm5350 | ENSMUSG00000098106 |
| chr9 | 85062119 | 85062141 | + | 4 | CAAAGTCATCAAAAAAGAAA | AGG | 42624 | Intergenic | Gm28070 | ENSMUSG00000101673 |
| chr17 | 81837589 | 81837611 | − | 4 | CAAAGTCATCAAAAAAGAAA | AGG | 45656 | Intergenic | Rpl31-ps25 | ENSMUSG00000097897 |
| chr15 | 46028134 | 46028156 | + | 4 | CAAAGTCATCAAAAAAGAAA | AGG | 48097 | Intergenic | Gm5472 | ENSMUSG00000096157 |
| chr11 | 8056844 | 8056866 | + | 4 | CAAAATCATCAAAAAAGAAG | AGG | 51828 | Intergenic | Gm24051 | ENSMUSG00000089247 |
| chrX | 109455183 | 109455205 | − | 4 | CAAAGTCATCAAAAAAGAAA | AGG | 63018 | Intergenic | Gm4784 | ENSMUSG00000082189 |
| chrX | 5542397 | 5542419 | − | 4 | CAAAGTCATCAAAAAAGAAA | AGG | 66153 | Intergenic | Gm14366 | ENSMUSG00000082326 |
| chr3 | 43476239 | 43476261 | + | 4 | CAAAGTCATCAAAAAAGAAA | AGG | 70912 | Intergenic | Gm36976 | ENSMUSG00000104303 |
| chr14 | 9098612 | 9098634 | + | 4 | CAAAGTCATCAAAAAAGAAA | AGG | NA | Intergenic | NA | NA |
| chrX | 17448219 | 17448241 | + | 4 | CAAAGTCATCAAAAAAGAAA | AGG | NA | Intergenic | NA | NA |
| chr8 | 54336974 | 54336996 | + | 4 | CAAAGTCATCAAAAAAGAAA | AGG | NA | Intergenic | NA | NA |
| chr8 | 52877304 | 52877326 | + | 4 | CAAAGTCATCAAAAAAGAAA | AGG | NA | Intergenic | NA | NA |
| chr8 | 51581196 | 51581218 | + | 4 | CAAAGTCATCAAAAAAGAAA | AGG | NA | Intergenic | NA | NA |
| chr5 | 40140103 | 40140125 | + | 4 | CAAAATCATCAAAAAAGAAA | AGG | NA | Intergenic | NA | NA |
| chr8 | 8159388 | 8159410 | + | 4 | CAAAGTCATCAAAAAAGAAG | AGG | NA | Intergenic | NA | NA |
| chr3 | 42706978 | 42707000 | − | 4 | CAAAGTCATCAAAAAAGAAA | AGG | NA | Intergenic | NA | NA |
| chr14 | 95475521 | 95475543 | − | 4 | CAAAGTCATCAAAAAAGAAA | AGG | NA | Intergenic | NA | NA |
| chr17 | 54775135 | 54775157 | − | 4 | CAAAGTCATCAAAAAAGAAA | AGG | NA | Intergenic | NA | NA |
| chr3 | 110832156 | 110832178 | − | 4 | CAAAGTCATCAAAAAAGAAA | AGG | NA | Intergenic | NA | NA |
| chrX | 143772054 | 143772076 | − | 4 | CAAACTTATCAAAAAAGAAA | AGG | 2286 | Intronic | Pak3 | ENSMUSG00000031284 |
| chr1 | 109798515 | 109798537 | + | 4 | CAAACTTATCAAAAAAGAAA | AGG | 6223 | Intergenic | Gm25293 | ENSMUSG00000084741 |
| chr4 | 37269996 | 37270018 | − | 4 | CAAACTTATCAAAAAAGAAA | AGG | 20438 | Intronic | Gm12374 | ENSMUSG00000086026 |
| chrX | 15343576 | 15343598 | + | 4 | CAAACTTATCAAAAAAGAAA | AGG | 21581 | Intergenic | Gm14519 | ENSMUSG00000082838 |
| chr4 | 78461286 | 78461308 | + | 4 | CACAGTTATCAAAAAAGAAA | AGG | NA | Intergenic | NA | NA |
| chr4 | 129153220 | 129153242 | − | 4 | CACACACCTCAAAAAAGAAG | AGG | 2129 | Intronic | S100pbp | ENSMUSG00000040928 |
| chr10 | 21936219 | 21936241 | + | 4 | CACACACGTCAAAAAAGAAA | GGG | 7668 | Intronic | Sgk1 | ENSMUSG00000019970 |
| chr7 | 5816912 | 5816934 | − | 4 | CAAACTTATCAAAAAAAGAG | AGG | 11467 | Intergenic | Vmn1r63 | ENSMUSG00000058631 |
| chr10 | 77348205 | 77348227 | − | 3 | CACAGTCATCAAAAAAAACA | AGG | 8029 | Intronic | Adarb1 | ENSMUSG00000020262 |
| chrX | 10517957 | 10517979 | + | 4 | GGCACTCATCAAAAAAAAGG | GGG | 7801 | Intronic | Gm25885 | ENSMUSG00000088404 |
| chr4 | 107057377 | 107057399 | − | 4 | CCCAGTCATCAAAAAAAAGG | TGG | 0 | Exonic | Mrpl37 | ENSMUSG00000028622 |
| chr7 | 86235583 | 86235605 | − | 4 | CAAAGTCATCAAAAAAAACA | AGG | 4313 | Intronic | Vmn2r76 | ENSMUSG00000091239 |
| chr6 | 60606333 | 60606355 | + | 4 | CAAAGTCATCAAAAAAAAGG | AGG | 15144 | Intergenic | RP24-89H23.1 | ENSMUSG00000108003 |
| chr2 | 65650992 | 65651014 | − | 4 | CAAAGTCATCAAAAAAAAGG | AGG | 17672 | Intronic | Scn2a1 | ENSMUSG00000075318 |
| chr7 | 125013982 | 125014004 | − | 4 | CAAAGTCATCAAAAAAAACA | AGG | 53025 | Intergenic | RP24-178G20. | ENSMUSG00000108407 |
| chr14 | 91164398 | 91164420 | − | 4 | CAAAGTCATCAAAAAAAAGA | AGG | 56106 | Intergenic | Gm23225 | ENSMUSG00000088746 |
| chr8 | 55580533 | 55580555 | + | 4 | CAAAGTCATCAAAAAAAACA | AGG | NA | Intergenic | NA | NA |
| chr9 | 16609244 | 16609266 | + | 4 | CAAAGTCATCAAAAAAAACA | AGG | NA | Intergenic | NA | NA |
| chr12 | 14607531 | 14607553 | − | 4 | CAAAGTCATCAAAAAAAACA | AGG | NA | Intergenic | NA | NA |
| chr11 | 95354796 | 95354818 | − | 4 | CACAGGCATCAAAAAAAAGC | GGG | 848 | Intronic | Fam117a | ENSMUSG00000038893 |
| chr8 | 119808110 | 119808132 | − | 4 | CACACACCTCAAAAAAAAGG | GGG | 1093 | Intergenic | Cotl1 | ENSMUSG00000031827 |

#P-4

Species: Mouse (Mus musculus GRCm38/mm10)

Input: TATTAGAAGAGCGAGGTCTGCGG

PAM: NGG

| Chromosome | start | end | strand | MM | target_seq | PAM | distance | position | gene name | gene id |
|---|---|---|---|---|---|---|---|---|---|---|
| chrX | 7579594 | 7579616 | + | 0 | TATTAGAAGAGCGAGGTCTG | CGG | 0 | Exonic | Ppp1r3fos | ENSMUSG00000039545 |
| chr7 | 50537428 | 50537450 | − | 3 | CTTTAGAAGAGTGAGGTCTG | TGG | 11490 | Intronic | Nell1os | ENSMUSG00000085265 |
| chr2 | 148559444 | 148559466 | + | 3 | GAATAGAAGAGAGAGGTCTG | AGG | 65245 | Intergenic | Gm25866 | ENSMUSG00000088870 |
| chr1 | 180703680 | 180703702 | − | 4 | GTTAAGAAGAGGGAGGTCTG | TGG | 6646 | Intergenic | Mixl1 | ENSMUSG00000026497 |
| chr5 | 35787005 | 35787027 | + | 4 | GGTGAGAAGAGAGAGGTCTG | GGG | 11426 | Intronic | Ablim2 | ENSMUSG00000029095 |
| chr3 | 79994232 | 79994254 | − | 4 | CAAGAGAAGAGGGAGGTCTG | CGG | 47952 | Intergenic | Fam198b | ENSMUSG00000027955 |
| chr9 | 21633138 | 21633160 | + | 3 | CATTAGAGGAGAGAGGTCTG | TGG | 84 | Intronic | Smarca4 | ENSMUSG00000032187 |
| chr5 | 28162360 | 28162382 | + | 4 | TGATAGAGGACCGAGGTCTG | CGG | 3312 | Intergenic | En2 | ENSMUSG00000039095 |
| chr5 | 91451610 | 91451632 | + | 4 | TGTGGGAAGAGAGAGGTCTG | AGG | 48616 | Intergenic | Btc | ENSMUSG00000082361 |
| chr14 | 19336816 | 19336838 | + | 4 | TTGTATAAGAGTGAGGTCTG | GGG | 64889 | Intergenic | Gm9637 | ENSMUSG00000093814 |
| chr4 | 114719100 | 114719122 | − | 4 | TGTTTTAAGAGTGAGGTCTG | GGG | 31505 | Intergenic | Gm12829 | ENSMUSG00000085870 |
| chr7 | 127304060 | 127304082 | + | 4 | GATCAGATGAGTGAGGTCTG | AGG | 851 | Intronic | Itgal | ENSMUSG00000030830 |
| chr14 | 29188009 | 29188031 | + | 4 | CTTTAGCAGAGCCAGGTCTG | GGG | 4368 | Intronic | Cacna2d3 | ENSMUSG00000021991 |
| chr3 | 153068414 | 153068436 | − | 3 | TATTAAAATAGTGAGGTCTG | AGG | 86168 | Intergenic | St6galnac5 | ENSMUSG00000039037 |
| chr10 | 118770781 | 118770803 | + | 4 | CTTTAGAAGTGTGAGGTCTG | AGG | 88546 | Intergenic | Dyrk2 | ENSMUSG00000028630 |
| chr6 | 134693269 | 134693291 | + | 4 | TACAAGAACAGTGAGGTCTG | AGG | 1967 | Intronic | RP23-246J18.! | ENSMUSG00000108166 |

| | | | | | | | | | | |
|---|---|---|---|---|---|---|---|---|---|---|
| chr18 | 30321996 | 30322018 | − | 4 | TGTTTGCAGAGCAAGGTCTG | TGG | 152 | Intronic | Pik3c3 | ENSMUSG00000033628 |
| chr8 | 72164293 | 72164315 | − | 4 | TTCTAGAAGACAGAGGTCTG | TGG | 2811 | Intronic | Rab8a | ENSMUSG00000003037 |
| chr17 | 24042572 | 24042594 | + | 4 | TGTTATAAGTGAGAGGTCTG | AGG | 208 | Intronic | Prss27 | ENSMUSG00000050762 |
| chr13 | 53496537 | 53496559 | − | 4 | TACTGGAAAAGCCAGGTCTG | CGG | 23463 | Intergenic | Msx2 | ENSMUSG00000021469 |
| chr13 | 109329044 | 109329066 | − | 3 | TATTAGATCAGCTAGGTCTG | AGG | 4220 | Intronic | Mir582 | ENSMUSG00000076946 |
| chr3 | 101664090 | 101664112 | + | 4 | TACTAGCAGGGAGAGGTCTG | AGG | 27677 | Intergenic | Gm43135 | ENSMUSG00000106070 |
| chr12 | 55856833 | 55856855 | + | 4 | TATTTTAGGAGCCAGGTCTG | AGG | 3236 | Intronic | Brms1l | ENSMUSG00000012076 |
| chr12 | 75019678 | 75019700 | + | 4 | TATTCAAGGAGCAAGGTCTG | TGG | 11879 | Intronic | Kcnh5 | ENSMUSG00000034402 |
| chr3 | 38669184 | 38669206 | + | 4 | TACTTGAAGAAGGAGGTCTG | GGG | 53626 | Intergenic | Gm43538 | ENSMUSG00000106168 |
| chr5 | 28602515 | 28602537 | − | 4 | CACTAGAAGAACAAGGTCTG | AGG | 23555 | Intronic | Gm43161 | ENSMUSG00000104587 |
| chr2 | 151531252 | 151531274 | − | 3 | TATTAAAAGAACAAGGTCTG | GGG | 9457 | Intergenic | Gm14167 | ENSMUSG00000087433 |
| chr3 | 59357028 | 59357050 | + | 4 | GGTTAGAAGAGGAAGGTCTG | TGG | 5861 | Intergenic | Gm38186 | ENSMUSG00000103668 |
| chr10 | 27937793 | 27937815 | − | 4 | GATGAGAAGAGAAAGGTCTG | GGG | 965 | Intergenic | Gm10145 | ENSMUSG00000064208 |
| chr2 | 146470509 | 146470531 | − | 4 | AATTAGAGGACCAAGGTCTG | AGG | 8846 | Intronic | Ralgapa2 | ENSMUSG00000037110 |
| chr19 | 42688813 | 42688835 | + | 4 | TATTACATGGGCAAGGTCTG | AGG | 10942 | Intergenic | Gm25216 | ENSMUSG00000077768 |
| chr9 | 44555490 | 44555512 | + | 4 | TGTTGGAAGAGAGTGGTCTG | GGG | 689 | Intronic | Cxcr5 | ENSMUSG00000047880 |
| chr6 | 120259486 | 120259508 | + | 3 | TCTAAGAAGAGCGAGGACTG | AGG | 16843 | Intronic | B4galnt3 | ENSMUSG00000041372 |
| chr13 | 92721717 | 92721739 | − | 4 | CATCAGAAGAACGACGTCTG | AGG | 9773 | Intergenic | Serinc5 | ENSMUSG00000021703 |
| chr1 | 71861152 | 71861174 | − | 4 | AATTAGCAGAGAGGGGTCTG | GGG | 14542 | Intergenic | Gm37217 | ENSMUSG00000102689 |
| chr2 | 4485517 | 4485539 | − | 4 | TTTAAGAAGAGCAGGGTCTG | AGG | 1385 | Intronic | Frmd4a | ENSMUSG00000026657 |
| chrX | 17203791 | 17203813 | − | 4 | AATTAGGGGAGCGAGCTCTG | TGG | 940 | Intronic | Efhc2 | ENSMUSG00000025038 |
| chr19 | 53344536 | 53344558 | + | 4 | CACTGGAAGAGCGAGGACTG | TGG | 1893 | Intronic | Mxi1 | ENSMUSG00000025025 |
| chr16 | 15890186 | 15890208 | − | 4 | TAATTGAAGAGGGAAGTCTG | GGG | 401 | Intronic | Spidr | ENSMUSG00000041974 |
| chr1 | 121947969 | 121947991 | + | 4 | CATTAAAAGAGGGAGTTCTG | TGG | 21226 | Intergenic | Gm15394 | ENSMUSG00000083584 |
| chr6 | 18215332 | 18215354 | − | 4 | TTGTAGAAGAGCCAGTTCTG | GGG | 1017 | Intronic | Cftr | ENSMUSG00000041301 |
| chr9 | 16178477 | 16178499 | − | 4 | TATAACAAGAGTGAGCTCTG | TGG | 68222 | Intronic | Fat3 | ENSMUSG00000074505 |
| chr6 | 3387973 | 3387995 | − | 4 | TATGAGATGAGCTATGTCTG | TGG | 2967 | Intronic | Samd9l | ENSMUSG00000047735 |
| chr6 | 3428507 | 3428529 | − | 4 | TATGAGATGAGCTATGTCTG | TGG | 28567 | Intergenic | Hepacam2 | ENSMUSG00000044156 |
| chr10 | 22886046 | 22886068 | − | 3 | TATGAGAAGAGGGAGGGCTG | AGG | 65918 | Intergenic | Tcf21 | ENSMUSG00000045680 |
| chr11 | 42358942 | 42358964 | + | 4 | TATAAGCAGAGCCAGTTCTG | GGG | 37870 | Intergenic | Gabra6 | ENSMUSG00000020428 |
| chr4 | 124861301 | 124861323 | − | 4 | TTTTAGGAGAGTGAGGCCTG | GGG | 310 | Intronic | Maneal | ENSMUSG00000042763 |
| chr7 | 67846934 | 67846956 | + | 4 | TATTGAAAGGGCGAGGTGTG | GGG | 43438 | Intergenic | 4833412C05Ri | ENSMUSG00000097697 |
| chr1 | 66693197 | 66693219 | − | 4 | GATGAGAAGAGAGAGGTGTG | AGG | 473 | Intronic | Unc80 | ENSMUSG00000055567 |
| chr12 | 108313546 | 108313568 | − | 4 | CATTAGGAGAGCCAGGTGTG | GGG | 211 | Intronic | 4930478K11Ri | ENSMUSG00000097847 |
| chr17 | 69282379 | 69282401 | + | 4 | TTTTAGATGAGCGAAGACTG | TGG | 1484 | Intronic | Epb41l3 | ENSMUSG00000024044 |
| chr13 | 64267636 | 64267658 | − | 4 | GTTTTGAAGAGCGAGGTCTC | AGG | 0 | Exonic | 1810034E14Ri | ENSMUSG00000097101 |
| chr13 | 40235613 | 40235635 | + | 4 | GCTTTGAAGAGCGAGGTCTT | GGG | 19803 | Intronic | Ofcc1 | ENSMUSG00000047094 |
| chr7 | 4597862 | 4597884 | + | 4 | TTTGAGAAGAGTGAGGTCAG | GGG | 30 | Intronic | Ptprh | ENSMUSG00000035429 |
| chr7 | 4573211 | 4573233 | + | 4 | TTTGAGAAGAGTGAGGTCAG | GGG | 30 | Intronic | Ptprh | ENSMUSG00000035429 |
| chr9 | 49718430 | 49718452 | + | 4 | GATAAGAAGAGCAAGGTCAG | AGG | 80226 | Intronic | Ncam1 | ENSMUSG00000039542 |
| chr5 | 20670367 | 20670389 | + | 4 | TATAAGGAGAGCGAGTGCTG | AGG | 9015 | Intronic | Magi2 | ENSMUSG00000040003 |
| chr4 | 106018041 | 106018063 | + | 4 | TAATAGATGAGAGAGGTCAG | AGG | 42156 | Intergenic | Gm24363 | ENSMUSG00000089065 |
| chr8 | 79987564 | 79987586 | − | 4 | TTTGAGAAGAACGAGGTCTA | GGG | 547 | Intronic | Hhip | ENSMUSG00000064325 |
| chr11 | 8521133 | 8521155 | + | 3 | TATTTGAAGAGCAAGGTCTC | AGG | 1563 | Intronic | Tns3 | ENSMUSG00000020422 |
| chr1 | 166996977 | 166996999 | − | 4 | TTTTATAAGAGGGAGGTCTC | TGG | 1296 | Intronic | Gm16701 | ENSMUSG00000097823 |
| chrX | 136525104 | 136525126 | + | 4 | TTTCAGAAGAGCCAGGTCTT | TGG | 0 | Exonic | Kir3dl1 | ENSMUSG00000031424 |
| chrX | 136457620 | 136457642 | − | 4 | TTTCAGAAGAGCCAGGTCTT | TGG | 0 | Exonic | Kir3dl2 | ENSMUSG00000057439 |
| chr1 | 59094382 | 59094404 | + | 4 | TATCAGATGAGGGAGGTCTC | AGG | 94 | Intronic | Als2cr11 | ENSMUSG00000072295 |
| chr14 | 14260183 | 14260205 | + | 4 | TATTATGAGAGCTAGGTCTC | AGG | 62705 | Intergenic | Olfr31 | ENSMUSG00000072707 |
| chr3 | 33805801 | 33805823 | − | 4 | TAATAGTAGAGCGAGGACTA | GGG | 993 | Intronic | Ttc14 | ENSMUSG00000027677 |
