# Supplementary material for: Stabilization of Foxp3 expression by CRISPR-dCas9-based epigenome editing in mouse primary T cells
Source: Epigenetics Chromatin. 2017 May 8;10:24. doi: 10.1186/s13072-017-0129-1 (PMC5422987; doi:10.1186/s13072-017-0129-1)
Supplement: Supplementary file 3 — Additional file 3: Figure S1. Experimental scheme of Foxp3 stability assay. Upper, naïve CD4+ T cells (CD4+CD62L+hCD2-) were MACS sorted and cultured under iTreg skewing conditions, and on day 2, dCas9-fusion protein and gRNA were transduced with polybrene. The next day, iTregs were harvested and further cultured under iTreg or inflammatory cytokine conditions for 2 days. Foxp3 expression (hCD2) was analyzed by flow cytometry. Lower, representative Foxp3 expression. Flow cytometry plots show expression of Foxp3 (endogenous) and Foxp3(hCD2, surface indicator) in primary T cells from Foxp3-hCD52-hCD2 KI mice. Figure S2. dCas9-TET1CD-mediated Foxp3 stabilization. (A) Histogram of Foxp3(hCD2) in dCas9-TET1CD (GFP/DsRed(+/-)) and dCas9-TET1CD with #C2-7 (GFP/DsRed(+/+)) cells under inflammatory conditions. Related to Figure 3b. (B) Foxp3 mRNA expression same as in Figure 3b. Data are pooled from three independent experiments and represent the means ± SDs. Figure S3. TGF-β signal enhanced effectiveness of dCas9-p300CD-mediated Foxp3 induction. Foxp3 expression induced by low-dose TGF-β in the presence of LY2157299 or anti-TGF-β was monitored by Foxp3(hCD2) MFI. Figure S4. dCas9-p300CD and gRNA co-transduced iTregs. (A) Sorting strategy and purification. (B) Suppression assay of iTregs comparing dCas9-p300CD and #P-4 with dCas9-p300CD catalytic mutant. [file 13072_2017_129_MOESM3_ESM.pdf]

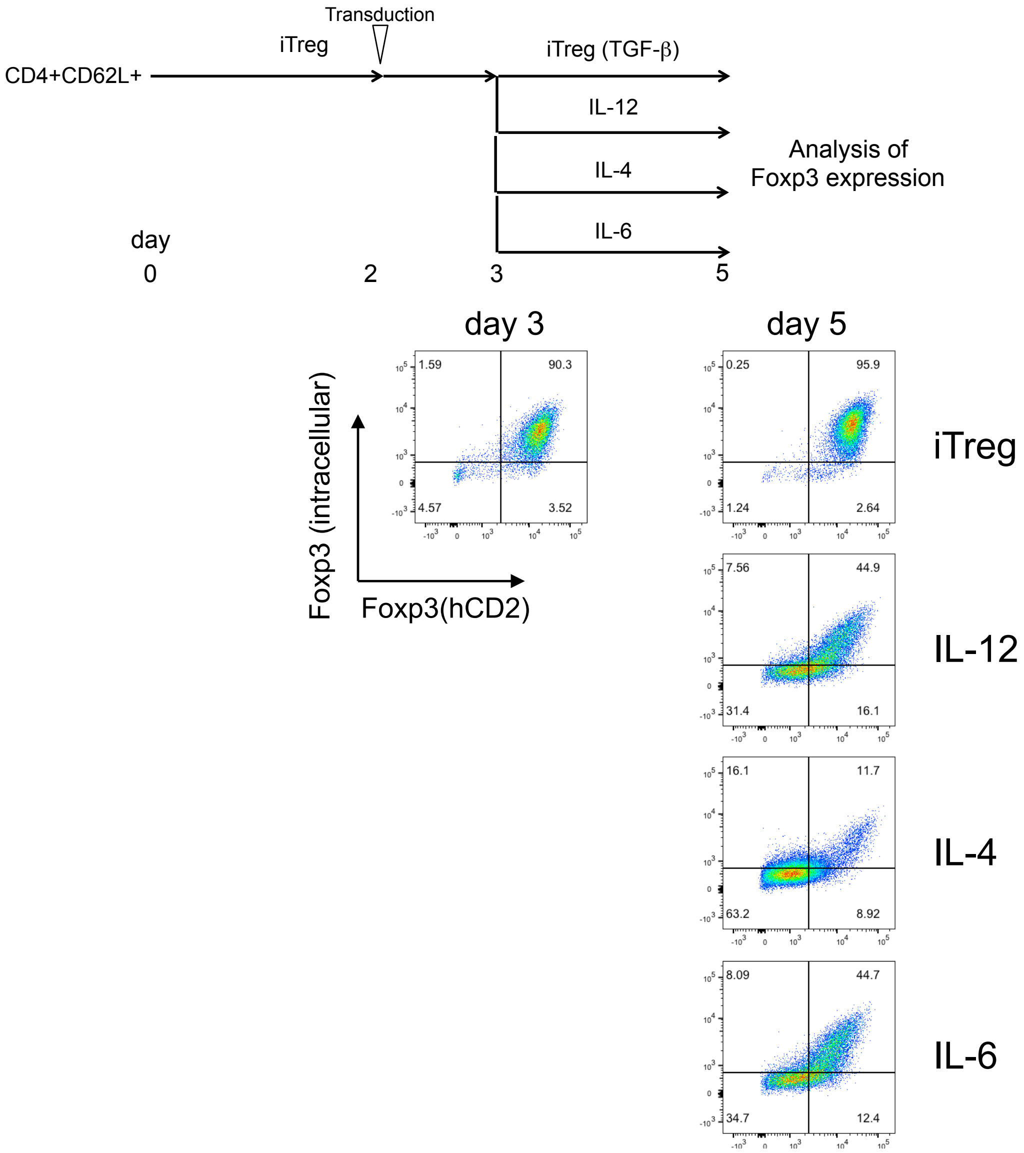

Supplementary Figure 1.
Okada et al.

a

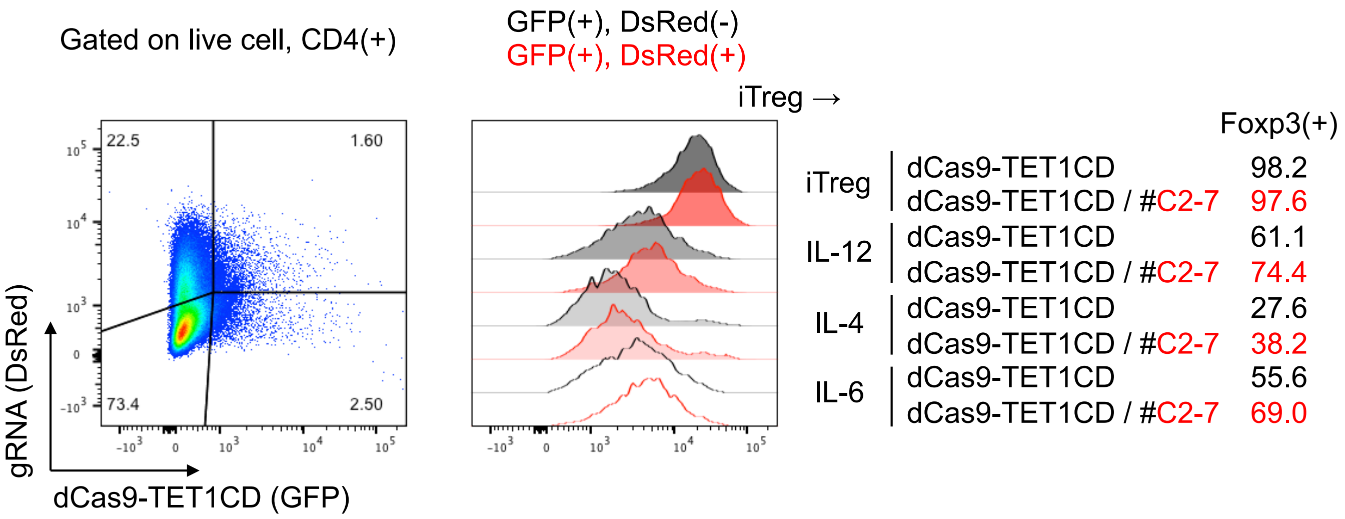

b

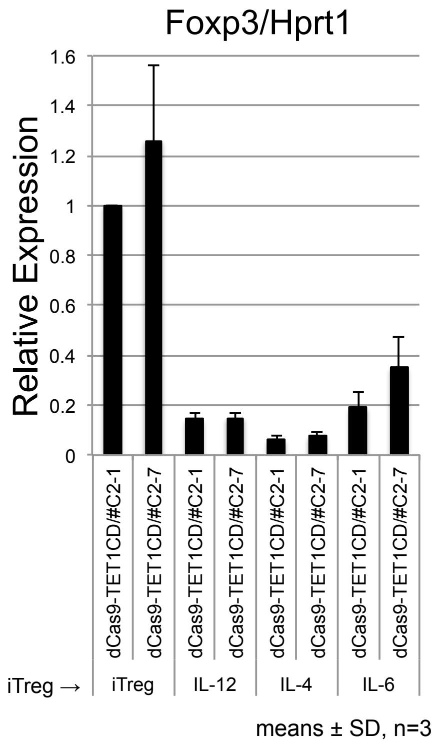

Supplementary Figure 2.
Okada et al.

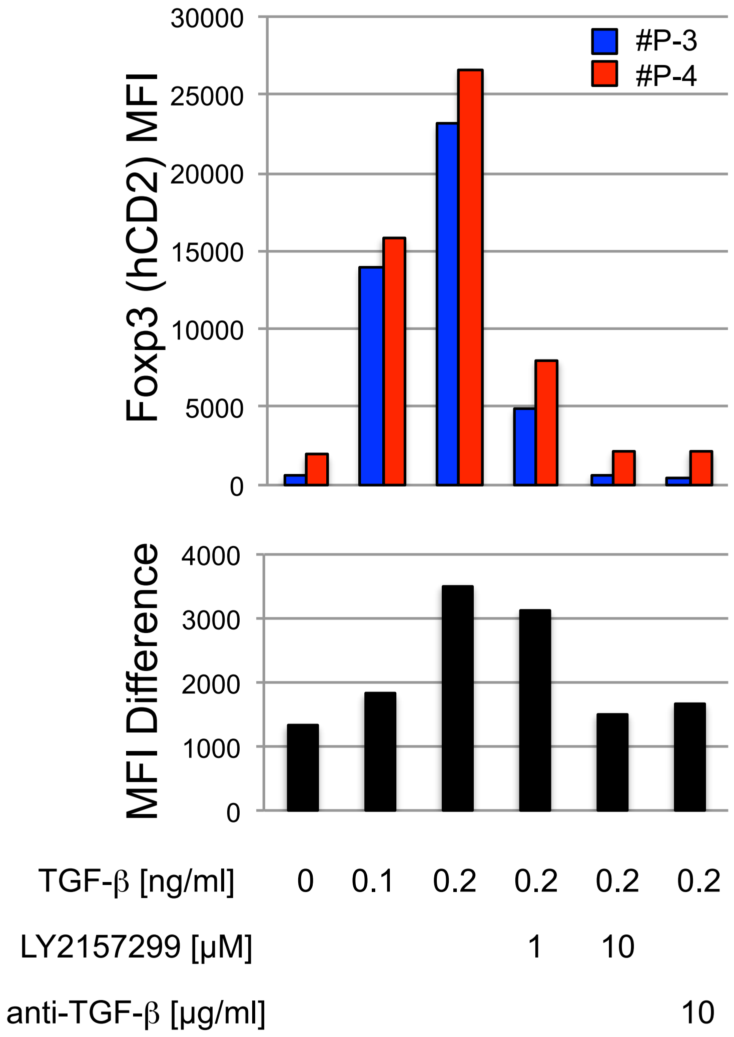

Supplementary Figure 3.
Okada et al.

a

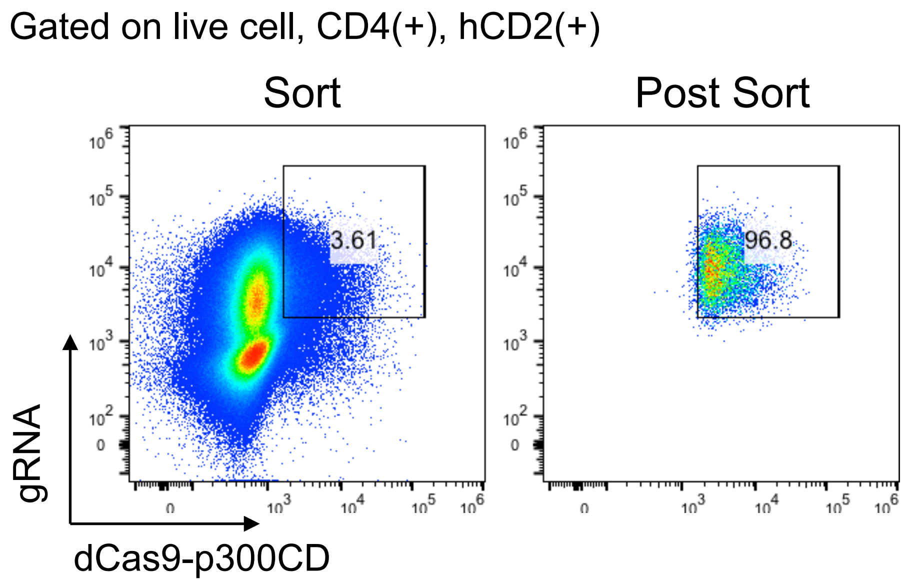

b

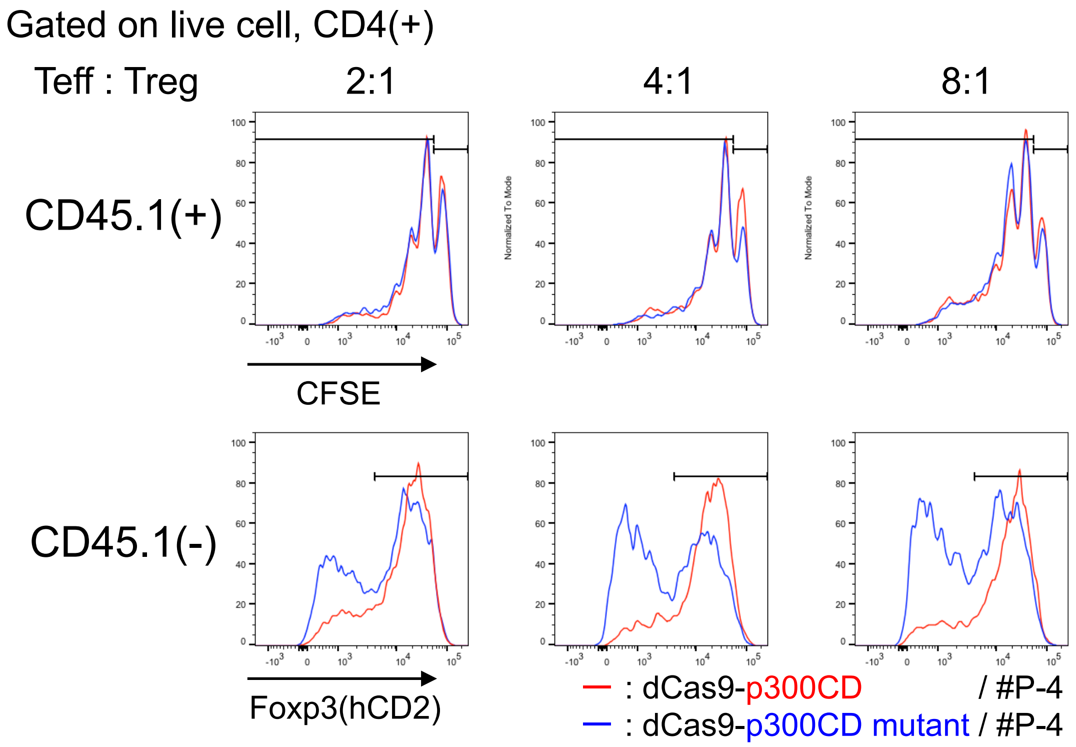

Supplementary Figure 4.
Okada et al.
